# Prognostic value of TIGIT in East Asian patients with solid cancers: A systematic review, meta-analysis and pancancer analysis

**DOI:** 10.3389/fimmu.2022.977016

**Published:** 2022-09-21

**Authors:** Sicong Li, Lanxing Li, Tianyan Pan, Xiaoqun Li, Yujia Tong, Yongdong Jin

**Affiliations:** ^1^School of Pharmacy, Peking University Health Science Centre, Beijing, China; ^2^School of Medicine, University of Electronic Science and Technology of China, Chengdu, China; ^3^Center of Disease Prevention Treatment, The Third Affiliated Hospital of Beijing University of Chinese Medicine, Beijing, China; ^4^Institute of Medical Information, Chinese Academy of Medical Sciences/Peking Union Medical College, Beijing, China; ^5^Department of Medical Oncology, Sichuan Cancer Hospital and Institute, Sichuan Cancer Center, School of Medicine, University of Electronic Science and Technology of China, Chengdu, China

**Keywords:** TIGIT, solid cancer, prognosis, meta-analysis, systematic review

## Abstract

**Background:**

T-cell immunoreceptor with Ig and ITIM domains (TIGIT) participates in tumor immune escape by delivering inhibitory signals to T cells. The purpose of this article was to assess the prognostic value of TIGIT and its immunological function in solid cancers.

**Methods:**

Three databases were searched for relevant articles. The main endpoints were overall survival (OS), progression-free survival (PFS), recurrence-free survival (RFS), and disease-free survival (DFS). Hazard ratios (HR) were pooled by using fixed-effects or random-effects models. Pancancer analysis of TIGIT was performed based on public online databases, mainly The Cancer Genome Atlas (TCGA), Genotype-Tissue Expression (GTEx), and UCSC Xena. The possible relationships between TIGIT expression and the tumor microenvironment (TME), infiltration of immune cells, immune-related genes, tumor mutation burden (TMB), and microsatellite instability (MSI) were revealed in this article.

**Results:**

Sixteen studies met the inclusion criteria. High expression of TIGIT was associated with worse OS [HR= 1.73, 95% confidence interval (CI) 1.50, 1.99], PFS (HR = 1.53, 95% CI [1.25, 1.88]), RFS (HR = 2.40, 95% CI [1.97, 2.93]), and DFS (HR= 6.57, 95% CI [0.73, 59.16]) in East Asian patients with solid cancers. TIGIT expression was positively correlated with immune infiltration scores and infiltration of CD8 T lymphocytes in all of the cancers included. TIGIT was found to be coexpressed with the genes encoding immunostimulators, immunoinhibitors, chemokines, chemokine receptors, and major histocompatibility complex (MHC), especially in gastroesophageal cancer. TMB and MSI were also associated with TIGIT upregulation in diverse kinds of cancers.

**Conclusion:**

High expression of TIGIT is associated with poorer prognosis in East Asian patients with solid cancers. TIGIT is a novel prognostic biomarker and immunotherapeutic target for various solid cancers because of its activity in cancer immunity and tumorigenesis.

## 1 Introduction

In the tumor microenvironment (TME), T cells are the second most abundant cell type after tumor-associated macrophages (TAMs) (1–3). Several immune inhibitor receptors (IRs), such as TIGIT, are upregulated in solid cancers and take part in tumor immune escape (4–6). As an important T-cell receptor in the TME, TIGIT competes with the costimulatory receptor cluster of differentiation 226 (CD226) for its interaction with the cluster of differentiation 155 (CD155) (7, 8) and participates in inhibiting adaptive and innate immunity. Highly expressed on active Regulatory T cells(Tregs), memory cluster of differentiation8(CD8) and memory cluster of differentiation (CD4) T-cell (9, 10), TIGIT can inhibit the cytotoxicity mediated by natural killer (NK) cells (1), the maturation and proinflammatory response of dendritic cells(DCs) (11), the effector functions of T helper cell 17(Th17) and T helper cell 1(Th1) cells (12), and enhance the immunosuppressive functions of Tregs by promoting the production of interleukin-10(IL-10) and fibrinogen-like protein 2 (Fgl2) (13).

The prognostic value of TIGIT has become a research hotspot in recent years, but the results remain controversial. Therefore, we conducted a meta- and bioinformatic analysis in this article for the following purposes: ①to evaluate the prognostic value of TIGIT in OS, DFS, PFS, and RFS and ② to determine the relationship between TIGIT expression and the tumor microenvironment and immune microenvironment.

## 2 Methods

### 2.1 Meta-analysis

#### 2.1.1 Data sources and search strategy

This systematic review and meta-analysis were performed according to the Preferred Reporting Items for Systematic Reviews and Meta-Analysis (PRISMA) guidelines (14). Embase (https://www.embase.com/), PubMed (https://pubmed.ncbi.nlm.nih.gov/), and the Cochrane Library (https://www.cochranelibrary.com/) were searched for articles. The retrieval time was from inception to May 28, 2022. This review was registered on the PROSPERO platform (CRD42022324498). The search strategy is described in **Supplementary Materials Tables 1–3**.

#### 2.1.2 Inclusion and exclusion criteria

Inclusion criteria included ① East Asian patients diagnosed with solid cancer before enrollment, ② randomized controlled trials (RCTs) or observational studies, ③ sufficient data about TIGIT expression and clinical outcome for meta-analysis, and ④ TIGIT expression was determined by using immunohistochemistry.

The exclusion criteria included ① case reports, single-cell sequencing data, animal experiments, meta-analyses, network meta-analyses, conference presentations, or study protocols.

#### 2.1.3 Outcomes

Outcomes included ①overall survival (OS), ② progression-free survival (PFS), ③ recurrence-free survival (RFS), and ④ disease-free survival (DFS).

#### 2.1.4 Study selection and data extraction

Two review authors (Sicong Li and Lanxing Li) independently reviewed the titles and abstracts of trials with potential eligibility. After that, we downloaded the full texts of trials eligible for inclusion. Two authors (Xiaoqun Li and Tianyan Pan) independently extracted the following data: ① basic information, including first author, publication year, sample size, country, and study design; ② characteristics of patients, including sex, age, type, and stage of cancer; ③ details about TIGIT, including expression location and cutoff value to judge high expression; ④ details about clinical outcomes; ⑤ information of cancer treatment; ⑥ information of quality assessment. Any disagreement was resolved by group discussion and consensus. We excluded results reported in only one study. In the studies that did not report HR values, we obtained the required data related to survival analysis from the survival curve by using GetData Graph Digitizer software.

#### 2.1.5 Strategy for meta-analysis

This meta-analysis was performed by using R (version 4.0.3). The chi-square test and ^χ2^ value were used to measure statistical heterogeneity. I2<50% and P value>0.05 indicated no substantial heterogeneity, and a fixed-effects model was used to pool the value of HR and 95% confidence interval. Otherwise, the random-effects model was used because of significant heterogeneity. Subgroup analysis was conducted to analyze sources of heterogeneity, while sensitivity analysis was conducted by excluding one study each time. Begg’s and Egger’s tests were used to assess publication bias. Statistical significance was set as α=0.05 in this study.

#### 2.1.6 Quality assessment

Two reviewers (Yongdong Jin, Yujia Tong) assessed the quality of eligible studies independently by using the Newcastle–Ottawa Quality Assessment Scale (NOS) (15). The NOS assessed the quality of studies from the aspects of selection, comparability, and exposure, with a total score ranging from 0 to 9 points. More than 6 points was defined as high-quality.

### 2.2 Pan-cancer analysis

#### 2.2.1 Data extraction and preprocessing

We downloaded the standardized pancancer data set from the UCSC (https://xenabrowser.net/) database: TCGA TARGET GTEx (PANCAN, N=19131, G=60499). Then, we extracted the expression data of the ENSG00000181847 (TIGIT) gene and 150 immune-related genes, including chemokines (n = 41), receptors (n = 18), major histocompatibility complexes (n = 21), immunoinhibitors (n = 24) and immunostimulators (n = 46), in normal solid tissues, primary solid tumors, normal tissues, primary blood-derived cancer-bone marrow, and peripheral blood. After excluding the cancer species with less than 3 samples in a single cancer species, the expression data of cancer species mentioned in the meta-analysis were finally obtained, including bladder urothelial carcinoma (BLCA), colon adenocarcinoma (COAD), colon adenocarcinoma/Rectum adenocarcinoma esophageal carcinoma (COADREAD), esophageal carcinoma (ESCA), liver hepatocellular carcinoma (LIHC), lung adenocarcinoma(LUAD), lung squamous cell carcinoma (LUSC), rectum adenocarcinoma (READ), stomach adenocarcinoma (STAD), skin cutaneous melanoma (SKCM), stomach and esophageal carcinoma(STES), thyroid carcinoma(THCA). We also extracted the gene expression profile of each tumor and converted the Tag names into gene symbols.

#### 2.2.2 Differential expression of TIGIT among tumor and normal samples

We used the unpaired Wilcoxon-rank sum and signed-rank tests to compare the difference in TIGIT expression between normal samples and tumor samples in each tumor. A violin plot was used to visualize the results.

#### 2.2.3 Differential expression of TIGIT among simple nucleotide variation (SNV) and copy number variation (CNV) data

From the GDC (https://portal.gdc.cancer.gov/) database, we downloaded the simple nucleoside variation (SNV) data set (level 4) and the copy number variation (CNV) data set (level 4) of all TCGA samples processed by MuTect2 (16) and GISTIC software (17), respectively. After removing samples of synonymous mutations, we obtained the expression data of 9 and 6 cancer species for CNV and SNV, respectively. Moreover, the domain information of TIGIT was obtained from the maftools package (version 2.2.10) of R software. A lollipop plot was used to depict the protein mutational distribution and domains.

#### 2.2.4 Relevance between TIGIT expression and the tumor microenvironment

We used the ESTIMATE package (version 1.0.13, https://bioinformatics.mdanderson.org/public software/estimate/) (18) to calculate the stromal, immune, and estimate scores for the cancers included in this article. The increased stromal and immune scores indicated an increased proportion of immune cells or stromal cells in the TME. Furthermore, the corr.test function of the psych package in R software (version 2.1.6) was used to conduct Pearson’s correlation test.

#### 2.2.5 Correlation between TIGIT expression and immune cell infiltration

We used the deconvo_CIBERSOR (19) and TIMER methods (20) in IOBR (version 0.99.9) (21) of R software to calculate the infiltration score of the 22 tumor-infiltrating immune cells, including naive B cells, memory B cells, plasma cells, CD8 T cells, naive CD4 T cells, resting and activated memory T cells, follicular helper T cells (Tfhs), regulatory T cells (Tregs), gamma delta T cells, resting and activated NK cells, monocytes, resting M0, M1 and M2 macrophages, resting and activated dendritic cells (DCs), resting and activated mast cells, eosinophils, and neutrophils. The results are displayed in the form of heatmap plots.

#### 2.2.6 Associations of TIGIT expression with immune-related genes, tumor mutation burden (TMB) level and microsatellite instability (MSI) status

First, we calculated the Spearman correlation between TIGIT and the 150 immunomodulators. The results were visualized in heatmap plots. Then, we used the TMB function of the maftools package (version 2.8.05) in R software to calculate the TMB score for each tumor and obtained the MSI score of each tumor from a previous study reported by Russell Bonneville (22). We integrated the MSI and gene expression data of the samples and further performed log2 (x+0.001) transformation on each expression value. The correlation between TIGIT expression and TMB or MSI was analyzed by means of the Spearman correlation coefficient, and the results are displayed in the form of lollipop plots (23).

#### 2.2.7 Protein−protein interaction network construction

GeneMANIA (http://www.genemania.org) was used to build a protein−protein interaction (PPI) network. Physical interaction, coexpression, and gene enrichment analyses were performed by using the network integration algorithm. The results were visualized by using the bioinformatic website (http://www.bioinformatics.com.cn/).

#### 2.2.8 Construction of competing endogenous RNA (ceRNA) networks

First, we used miRwalk (http://mirwalk.umm.uni-heidelberg.de/accessed on 30 May 2022) to predict target miRNAs of TIGIT that can bind to the TIGIT 3′-UTR. Next, miRNA–lncRNA interactions were obtained from RNAInter v4.0 (http://www.rnainter.org/search/) with the species set as Homo sapiens. Finally, the ceRNA network was visualized by using Cytoscape 3.8.2 (24) software.

#### 2.2.9 Statistical analysis

Statistical data analysis was conducted by using R software (version 4.0.3) (https://www.r-project.org/). The unpaired Wilcoxon-rank sum and signed-rank tests were used to analyze the significance of the difference between two groups, and the Kruskal test was used to test the difference among multiple groups of samples. The correlation between TIGIT expression and the other variable was assessed utilizing the Spearman correlation coefficient. P values of less than 0.05, 0.01 and 0.001 are presented as “*”, “**”, and “***”, respectively.

## 3 Results

### 3.1 Results of the meta-analysis

#### 3.1.1 Search results

**Figure 1:**
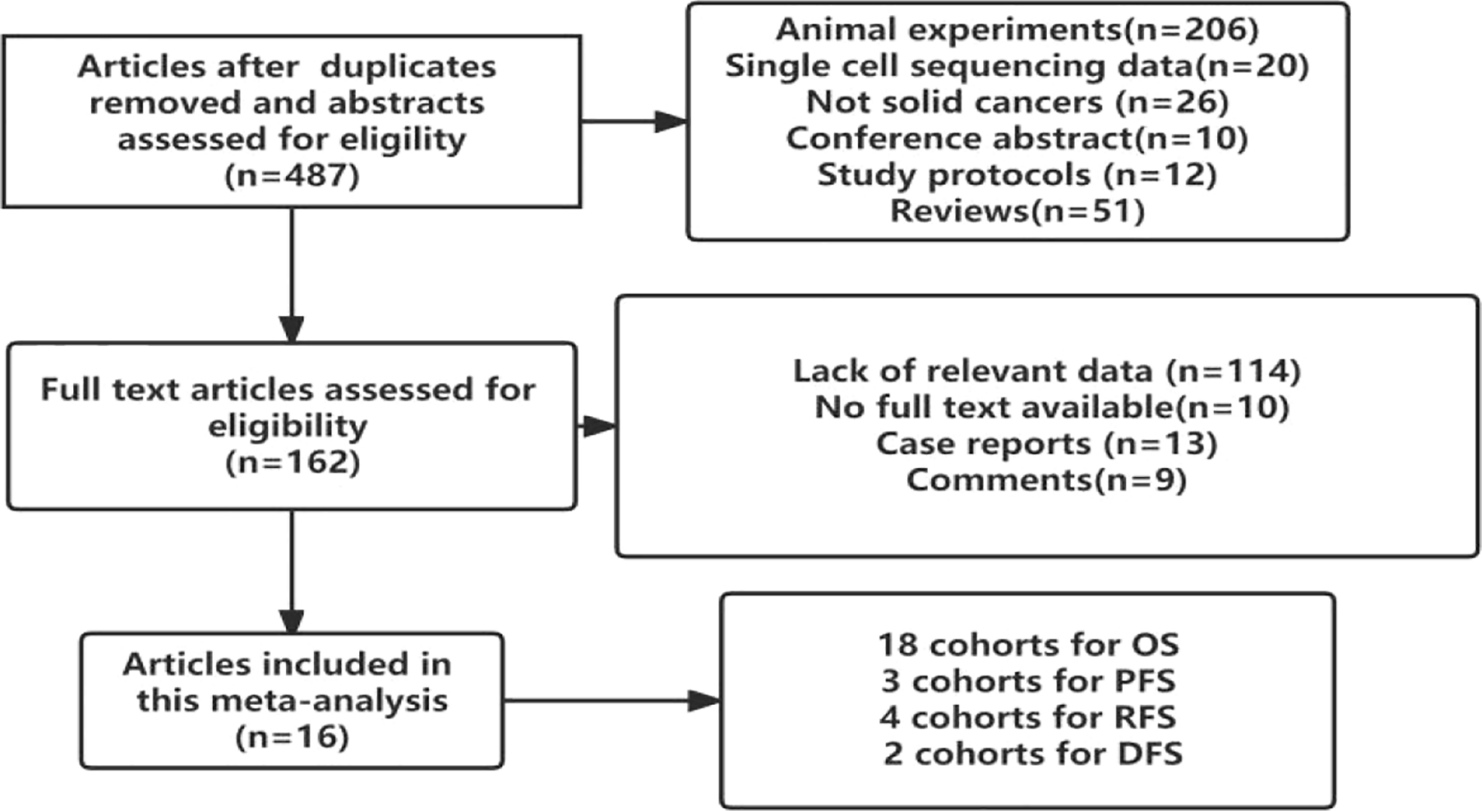
Study selection flowchart.

In total, 16 studies involving 2488 patients with solid cancers were found to meet the inclusion criteria. All of them were retrospective cohort studies. Although Pooja Ghatalia (25) and Pankaj Ahluwalia (26) reported the prognostic value of TIGIT in cancer patients, they did not report the hazard ratio or odds ratio value of TIGIT. Therefore, the patients included were from the East Asian population. The flow chart of the study selection process is presented in **Figure 1**.

#### 3.1.2 Study characteristics

The basic characteristics of the included studies are shown in **Table 1**. Studies were published between 2018 and 2022. Zhao JJ (27), Peipei Wang (28) and Zhao K (29) reported survival outcomes of esophageal squamous cell carcinoma (ESCC) or primary small cell carcinoma of esophagus (PSCCE), Tang W (30) and Liu HF (31) reported gastric cancer (GC), Xu Y (32) reported small cell lung cancer (SCLC), Sun Y (33) and Jiang C (34) reported non-small cell lung cancer (NSCLC), Zhou X (35), Liang R (36) and Daisuke Murakami (37) reported colorectal cancer (CRC), Lee WJ (38) reported melanoma, Yu LH (39) reported hepatitis B virus hepatocellular carcinoma (HBV-HCC), and Liu ZP et al. reported the ZSHS cohort and the FUSCC cohort that reported survival outcomes of patients with muscle-invasive bladder cancer (MIBC) (40). Luo Y ^reported^ (41) 3 cohorts of patients with advanced thyroid carcinoma (ATC), including anaplastic thyroid carcinoma (ATC), poorly differentiated thyroid carcinoma (PDTC), and locally advanced papillary thyroid carcinoma (PTC). HR values in the studies reported by Xu Y (2019), Liang R (2021), Lee WJ (2020), Sun Y (2020), and Zhao K (2020) were obtained by calculating the data extracted from the survival curve.

**Table 1:** Basic characteristics.

Author	Sample size	Country	Age	male/female	Cancer	Treatment other than surgery	TIGIT+ expression	Expression location	Cutoff value of TIGIT	Outcome	Method to estimate HR
Zhao JJ (2018)	154	China	55 (37–48)	(124/30)	ESCC	Not available	76 (49.4%)	TIL	Median level	OS	Multivariate
Tang W(2019)	441	China	(<62:159, ≥62:282)	(245/196)	GC	Adjuvant chemotherapy	343 (77.8%)	Tumor cell	≥5% positivity cell	OS	Multivariate
Xu Y(2019)	60	China	(≤60:34,>60:26)	(43/17)	SCLC	Adjuvant chemotherapy	21 (35%)	Tumor cell	Median level	OS	Univariate
Lee WJ (2020)	124	Korea	61.8(25-89)	(68/56)	Melanoma	Not available	52 (41.9%)	Tumor cell	≥20% positivity cell	OS/PFS	Univariate
Sun Y (2020)	334	China	56(28-81)	(182/152)	NSCLC	Not available	204 (61.1%)	TIL	≥5% positivity cell	OS/PFS	Multivariate
Zhao K (2020)	114	China	≤60 76, >60 38	(84/30)	PSCCE	Adjuvant chemotherapy/chemoradiotherapy.	74 (64.9%)	Tumor cell	≥5% positivity cell	OS/PFS	Multivariate
Zhou X (2020)	60	China	≤60 34, >60 26	(35/25)	CRC	Not available	21 (35%)	Tumor cell	CPS≥1	OS/DFS	Multivariate
Liang R(2021)	139	China	≤45:25, >45 114	(82/57)	CRC	Not available	40 (28.8%)	Tumor cell	Median level	OS/RFS	Univariate
Liu HF (2022)	194	China	56 ± 12.66	(135/59)	GC	Adjuvant chemotherapy	97(50%)	TIL	Median level	OS	Multivariate
Peipei Wang (2021)	95	China	58 ± 10	(81/14)	ESCC	Not available	68(72%)	Tumor cell	Median level	OS	Multivariate
Daisuke Murakami(2022)	100	Japan	> 70 53 (53%); < 70 years 47 (47%)	(55/45)	CRC	Not available	79(79%)	TIL	≥10% positivity cell	OS	Multivariate
Jiang C (2022)	81	China	63(29–81)	(68/13)	NSCLC	Not available	33 (40.7%)	TIL	CPS≥1	OS	Multivariate
Yu LH (2021)	133	China	58.3 ± 11.4	(103/30)	HBV-HCC	Adjuvant immunotherapy ± adjuvant chemotherapy	65(48.87%)	TIL	Median level	PFS	Multivariate
Shi X (2021)	200	China	49 (12–80)	(105/95)	MTC	Not available	6 (3.0%)	Tumor cell	CPS≥1	OS/RFS	Univariate
Liu ZP (2020)	141	China	ZSHC cohort 62(56-71);FUSCC cohort 62(56-68)	ZSHC cohort (117/24);FUSCC cohort(102/16)	MIBC	Adjuvant chemotherapy	ZSHC cohort46(32.62%);FUSCC cohort 68(57.63%)	TIL	Median level	OS/RFS	Multivariate
Luo Y (2022)	234	China	55.50 (41.25, 66.50)	112(47.86%)	TC	Not available	37(15.81%)	Tumor cell and TIL	CPS≥1	OS/DFS	Multivariate

The HR values in the studies reported by Xu Y (2019), Liang R (2021), Lee WJ (2020), and Shi X (2021) were estimated by only univariate analysis, while the others were estimated by both multivariate analyses. Regarding the quality of the included studies, NOS scores ranged from 6 to 8.

#### 3.1.3 Results of OS, PFS, RFS, and DFS

**Figure 2:**
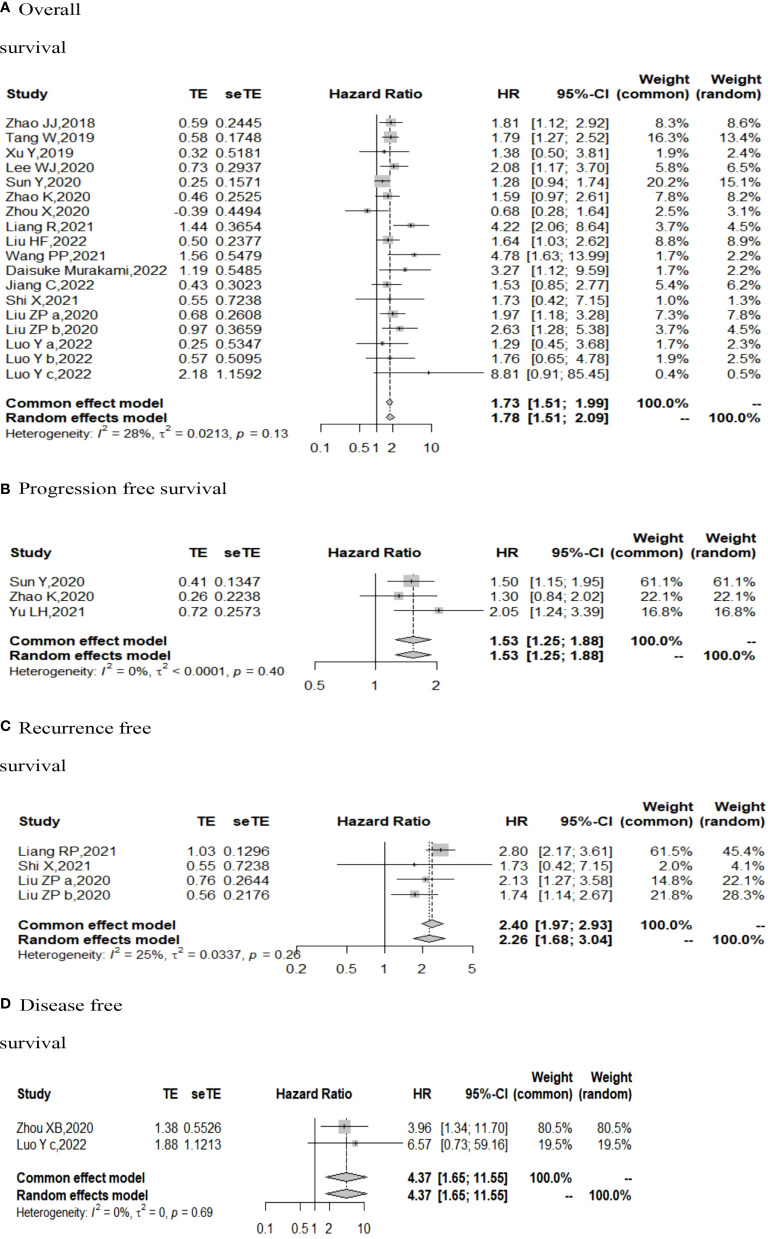
Forest plot for all outcomes. (**A**. Overall survival **B**. Progression free survival **C**. Recurrence free survival **D**. Disease free survival).

The I (2) value was less than 50%, and the p value was above 0.05, so the fixed-effect model was used in the comparison of OS, PFS, RFS, and DFS. The pooled results of the meta-analysis showed that high expression of TIGIT was associated with shorter OS (HR = 1.73, 95% CI [1.50, 1.99]), PFS (HR =1.53, 95% CI[1.25,1.88]), RFS (HR = 2.40, 95% CI [1.97, 2.93] and DFS (HR = 4.37, 95% CI [1.65, 11.55]) in patients with solid cancers than low expression of TIGIT (see **Figure 2**).

**Figure 3:**
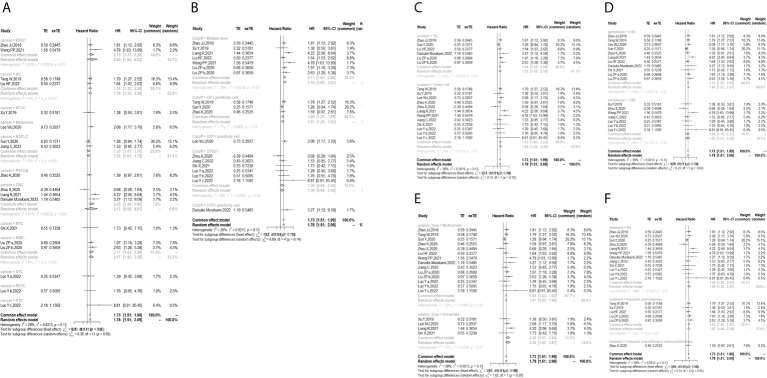
Subgroup analysis for the relationship between TIGIT and overall survival **(A)** Grouped by different cancers **(B)** Grouped by different cutoff values of TIGIT expression **(C)** Grouped by location of TIGIT expression **(D)** Grouped by sample size **(E)** Grouped by different methods to estimate HR **(F)** Grouped by different postoperative treatments).

**Figure 4:**
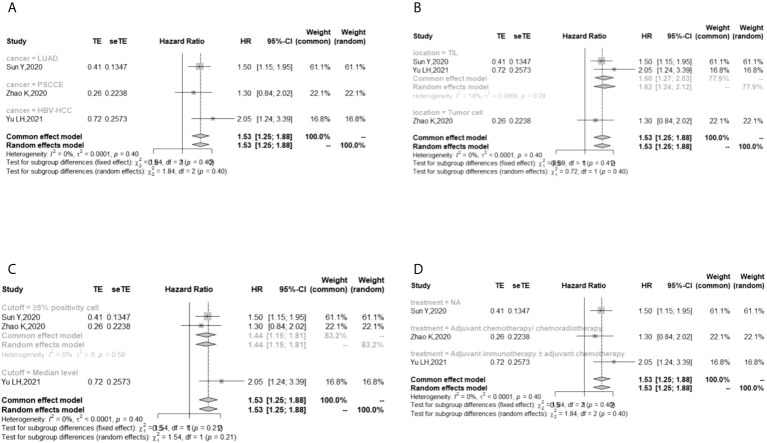
Subgroup analysis for the relationship between TIGIT and progression-free survival **(A)** Grouped by different cancers **(B)** Grouped by location of TIGIT expression **(C)** Grouped by different cutoff values of TIGIT expression **(D)** Grouped by different postoperative treatments).

**Figure 5:**
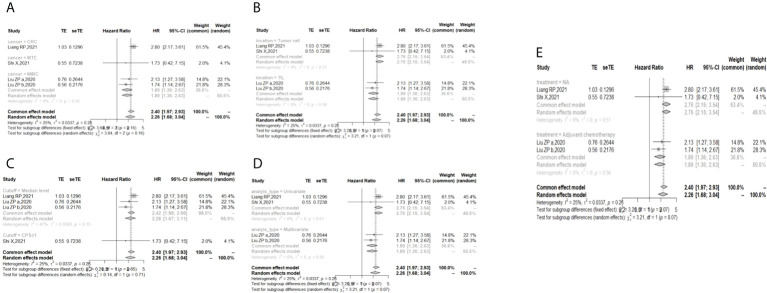
Subgroup analysis for the relationship between TIGIT and recurrence-free survival **(A)** Grouped by different cancers **(B)** Grouped by location of TIGIT expression **(C)** Grouped by different cutoff values of TIGIT expression **(D)** Grouped method to estimate HR **(E)** Grouped by different postoperative treatments).

**Figure 6:**
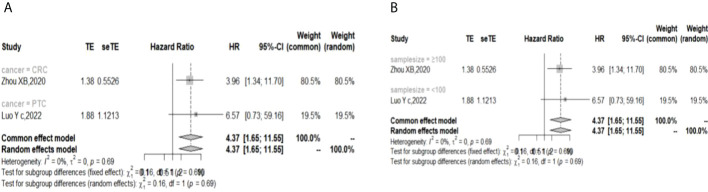
Subgroup analysis for the relationship between TIGIT and disease-free survival **(A)** Grouped by different cancers **(B)** Grouped by different sample sizes).

In the 2 studies about DFS, TIGIT was expressed on tumor cells, and CPS≥1 was set as the cutoff value of TIGIT expression. Moreover, multivariate analysis was used to estimate the HR value in the comparison of DFS and PFS, so we did not conduct subgroup analysis in these aspects. No significant prognostic value of TIGIT was found in the OS of cancers, including SCLC, CRC, MTC, ATC, PDTC and PTC. Studies with sample sizes <100 did not support the prognostic value of TIGIT and OS (HR = 1.55, 95% CI [0.76, 3.19]). PSCCE and TIGIT expressed on tumor cells did not support the prognostic value of TIGIT in PFS, while medullary thyroid carcinoma and papillary thyroid carcinoma did not support the prognostic value of TIGIT in RFS and DFS, respectively. Postoperative treatments were not found to be a source of heterogeneity in OS and RFS, probably because nearly half of the studies did not describe them. In terms of PFS, adjuvant chemotherapy/chemoradiotherapy did not support the prognostic value of TIGIT, and this result should be discussed with care because of limited study numbers and sample sizes (see **Figures 3**–**6**).

The p values of Begg’s test and Egger’s test for OS and RFS were above 0.05, which indicated no significant publication bias. In the sensitivity analysis, the DFS results would change if Zhou XB (2020) was omitted. (Liang R, 2021), (Yu LH,2021), (Liu ZP, 2020), and (Luo YC,2022) contributed the most to the overall heterogeneity in OS, PFS, RFS, and DFS, respectively.

### 3.2 Pancancer analysis

**Figure 7:**
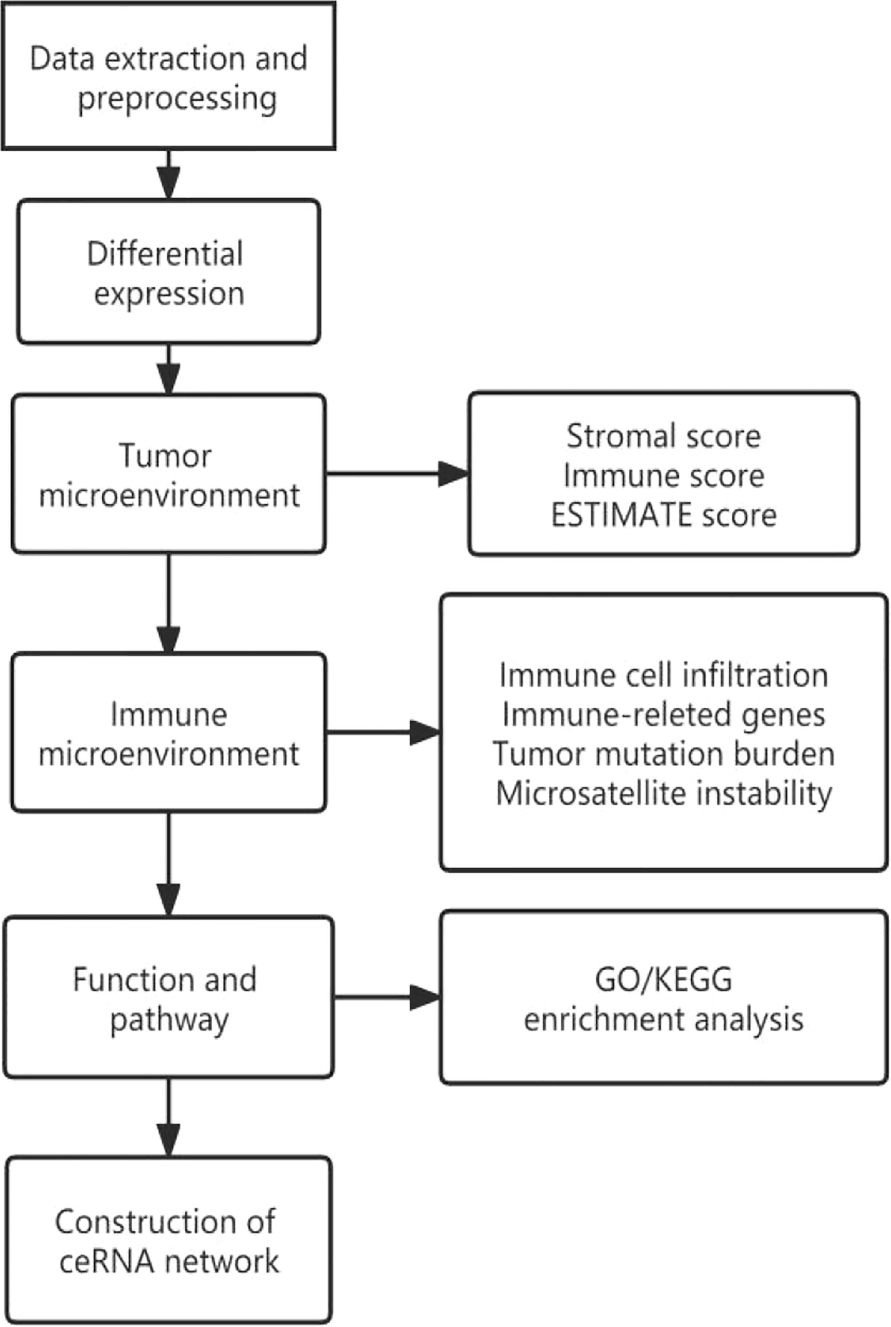
Flow chart of pancancer analysis.

The design flow and implementation approaches of this study are illustrated in **Figure 7**. This study integrally revealed the role of TIGIT in the tumor immune microenvironment.

#### 3.2.1 Differential expression of TIGIT among cancer and normal samples

**Figure 8:**
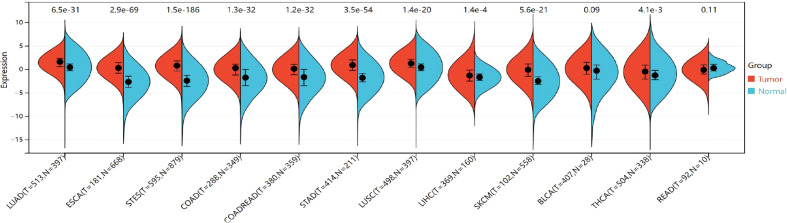
Differential TIGIT mRNA expression in 12 kinds of cancers and normal tissues by using the combination of box diagram and violin diagram. P values were presented by using scientific notation. LUAD, lung adenocarcinoma; ESCA, esophageal carcinoma; STES, Stomach and Esophageal carcinoma; COAD, colon adenocarcinoma; COADREAD, colon adenocarcinoma/Rectum adenocarcinoma esophageal carcinoma; STAD stomach adenocarcinoma; LUSC, Lung squamous cell carcinoma; LIHC, liver hepatocellular carcinoma; SKCM, skin cutaneous melanoma; THCA, hyroid carcinoma; READ, rectum adenocarcinoma.

The expression of TIGIT was significantly upregulated in the 9 cancers involved in this study, including LUAD (tumor:1.44 ± 1.33, normal:0.49 ± 0.99, P=2.6e^-32^<0.05), ESCA (tumor: 0.29 ± 1.59, normal:-2.58 ± 1.72,P=2.5e^-57^<0.05), STES (tumor: 0.71 ± 1.57, normal: -2.35 ± 1.73, P=1.3e^-151^<0.05), COAD (tumor:0.01 ± 1.62,normal:-1.81 ± 2.07, P=1.4e^-26^<0.05), COADREAD (tumor:-0.03 ± 1.60,normal:-1.75 ± 2.08,P=1.9e^-27^<0.05), STAD (tumor:0.89 ± 1.53,normal:-1.66± 1.58,P=7.8e^-54^<0.05),LUSC (tumor:1.25 ± 1.36,normal:0.49 ± 0.99,P=1.8e^-22^<0.05),LIHC (tumor:-1.18 ± 1.86,normal:-1.71 ± 1.23,P=3.4e^-3^<0.05),SKCM (tumor:-0.10 ± 2.07,normal:-2.40 ± 1.31,P=1.6e^-26^<0.05),THCA (tumor:-0.51 ± 2.01,normal:-0.89 ± 1.92,P=1.6e^-4^<0.05). However, no significant difference was observed between cancer and normal samples in BLCA (P=0.09>0.05) and READ (P=0.11>0.05) (**Figure 8**). The TIGIT expression values in different cancers are shown in **Supplementary Table 5**.

#### 3.2.2 Impact of CNV and SNV on TIGIT expression

TIGIT expression was higher in CNV neutrals than in CNV gains in STAD and LUSC. Moreover, TIGIT expression was higher in CNV neutral samples than in samples with CNV losses in LIHC and SKCM. This result indicated that CNV influenced TIGIT expression. No significant difference was found in TIGIT expression among patients with wild type and TIGIT mutation in terms of SNV.

**Figure 9:**
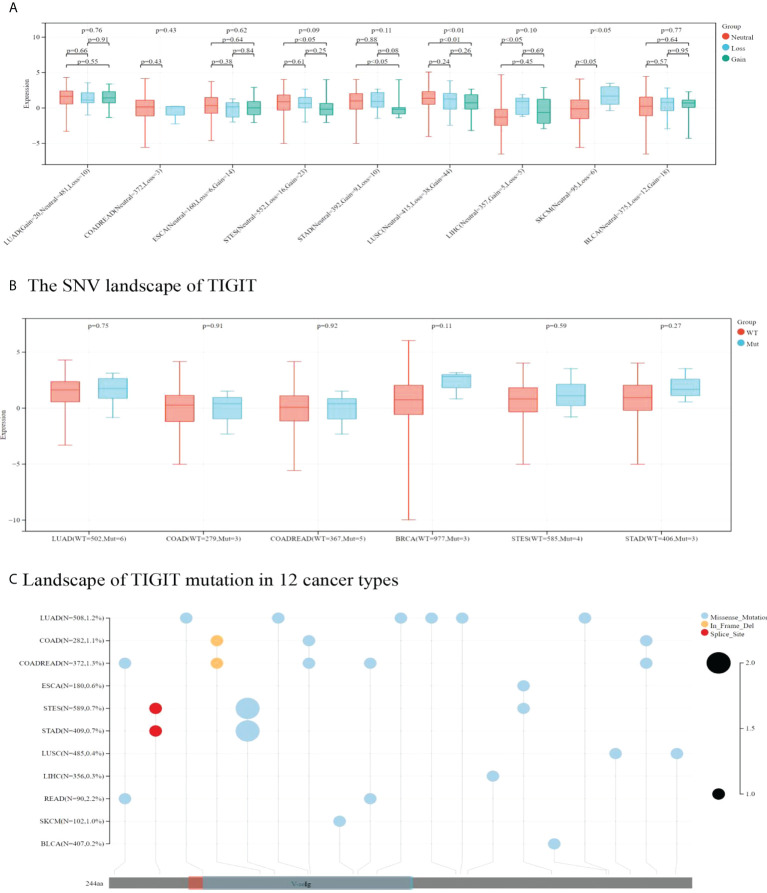
In **A** and **B** correlation between TIGIT expression and CNV and SNV were presented in boxplot. P values were presented by using scientific notation. In **C**, different color patches represent different domains of TIGIT. The sites marked with lollipops are mutation sites. Circular size represents mutation frequency.

Missense mutation was the most common type of mutation in the cancers included. Missense mutations in V-seIg were found in STES, COAD, COADREAD, LUAD, STAD, SKCM and READ. Among them, LUAD was found to have the most missense mutations. In-frame deletion occurred in COAD and COADREAD. A splice site was found in STAD and STES (see **Figure 9**). READ presented a relatively high mutation frequency (2.2%). TIGIT expression values in patients with wild-type, SNV or CNV are presented in **Supplementary Tables 6, 7**.

#### 3.2.3 Correlation between TIGIT expression and the tumor microenvironment

Stromal and immune cells are two main types of nontumor components in the TME. The ESTIMATE algorithm can help to predict the tumor purity in tumor samples. We calculated the Spearman correlation coefficient between TIGIT expression and immune infiltration scores by using corr.test psych (version 2.1.6) in R software. Finally, a significant positive correlation was observed between TIGIT expression and immune infiltration scores (Stromals score, Immunes score and ESTIMATE score) in 12 kinds of cancers involved in these studies (see **Supplementary Figures 5–7**).

#### 3.2.4 Correlation between TIGIT expression and the infiltration score of immune cells (CIBERSORT AND TIMER)

**Figure 10:**
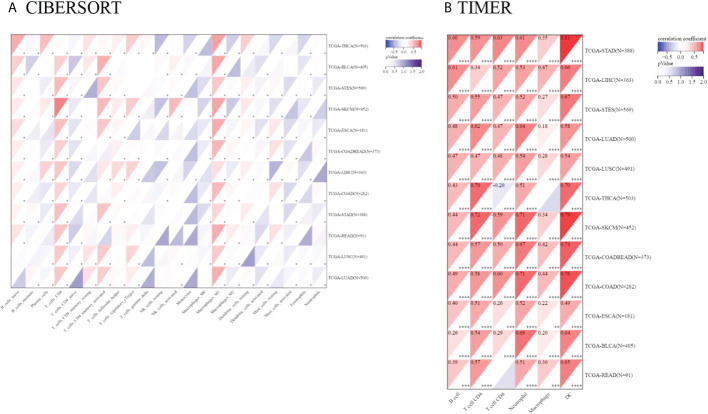
Pancancer analysis of the Spearman correlation between TIGIT expression and immune cell infiltration. CIBERSORT in **(A)**, TIMER in **(B)**. Red represents a positive correlation, and blue represents a negative correlation. The darker the color is, the greater the correlation coefficient. ^∗^P< 0.05, ^∗∗^P< 0.01, ^∗∗∗^P< 0.001 and ^∗∗∗∗^p< 0.0001.

Tumor-infiltrating immunocytes could affect the survival prognosis of patients. TIGIT expression showed a positive correlation with the infiltration scores of CD8 T cells (especially in SKCM), M1 macrophages in 12 kinds of cancers, naive B cells in 10 kinds of cancers, activated memory CD4 T cells, Tregs in LUAD, LUSC, LIHC, SKCM, THCA, COADREAD and activated NK cells in LUAD, LUSC, SKCM, STES, BLCA. TIGIT expression was also negatively correlated with activated dendritic cells and mast cells in most of the cancers included. The correlation coefficient between TIGIT expression and immune cell infiltration (CIBERSORT) is presented in **Supplementary Table 8**. Moreover, TIGIT expression was found to be positively related to B cell, CD4, CD8 T cell, neutrophil, macrophage and DC infiltration in most cancers based upon the TIMER algorithm (**Figure 10**).

#### 3.2.5 Correlation between TIGIT expression and 150 immune-related genes

**Figure 11:**
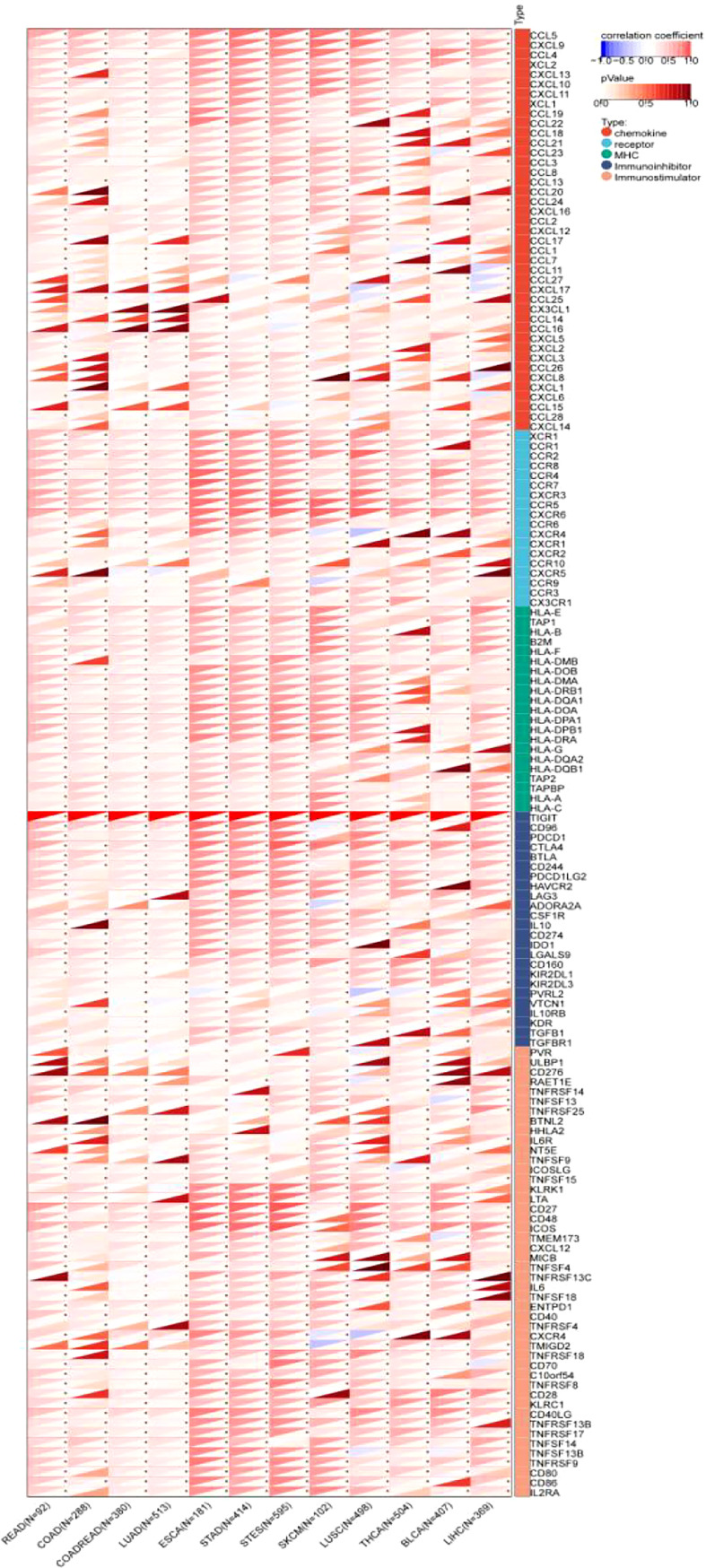
Pancancer analysis of the Spearman correlation betweenTIGIT expression and 150 immune-related genes, including 41 chemokines, 24 immunoinhibitors, 46 immunostulators, 21 MHCs and 18 receptors. Red represents a positive correlation, and blue represents a negative correlation. The darker the color is, the greater the correlation coefficient. ^∗^P< 0.05, ^∗∗^P< 0.01, and ^∗∗∗^P< 0.001. CCL5, C-C Motif Chemokine Ligand 5; CXCL9, C-X-C Motif Chemokine Ligand 9; CCL4, C-C Motif Chemokine Ligand 4;XCL2, X-C Motif Chemokine Ligand 2; CXCL13, C-X-C Motif Chemokine Ligand 13; CXCL10, C-X-C Motif Chemokine Ligand 10; CXCL11, C-X-C Motif Chemokine Ligand 11; XCL1, X-C Motif Chemokine Ligand 1; CCL19, C-C Motif Chemokine Ligand 19; CCL22, C-C Motif Chemokine Ligand 22; CCL18, C-C Motif Chemokine Ligand 18; CCL21, C-C Motif Chemokine Ligand 21; CCL23, C-C Motif Chemokine Ligand 23; CCL3, C-C Motif Chemokine Ligand 3; CCL8, CC Motif Chemokine Ligand 8; CCL13, C-C Motif Chemokine Ligand 13; CCL20, C-C Motif Chemokine Ligand 20; CCL24, C-C Motif Chemokine Ligand 24; CXCL16, C-X-C Motif Chemokine Ligand 16; CCL2, C-C Motif Chemokine Ligand 2; CXCL12, C-X-C Motif Chemokine Ligand 12; CCL17, C-C Motif Chemokine Ligand 17; CCL1, C-C Motif Chemokine Ligand 1; CCL7, C-C Motif Chemokine Ligand 7; CCL11, C-C Motif Chemokine Ligand 11; CCL27, C-C Motif Chemokine Ligand 27; CXCL17, C-X-C Motif Chemokine Ligand 17; CCL25, C-C Motif Chemokine Ligand 25; CX3CL1, C-X3-C Motif Chemokine Ligand 1; CCL14, C-C Motif Chemokine Ligand 14; CCL16, C-C Motif Chemokine Ligand 16; CXCL5, C-X-C Motif Chemokine Ligand 5; CXCL2, C-X-C Motif Chemokine Ligand 2; CXCL3, C-X-C Motif Chemokine Ligand 3; CCL26, C-C Motif Chemokine Ligand 26; CXCL8, C-X-C Motif Chemokine Ligand 8; CXCL1, C-X-C Motif Chemokine Ligand 1; CXCL6, C-X-C Motif Chemokine Ligand 6; CCL15, C-C Motif Chemokine Ligand 15; CCL28, C-C Motif Chemokine Ligand 28; CXCL14, C-X-C Motif Chemokine Ligand 14; XCR1, X-C Motif Chemokine Receptor 1; CCR1, C-C Motif Chemokine Receptor 1; CCR2, C-C Motif Chemokine Receptor 2; CCR8, C-C Motif Chemokine Receptor 8; CCR4, C-C Motif Chemokine Receptor 4; CCR7, C-C Motif Chemokine Receptor 7; CXCR3, C-X-C Motif Chemokine Receptor 3; CCR5, C-C Motif Chemokine Receptor 5; CXCR6, C-X-C Motif Chemokine Receptor 6; CCR6, C-C Motif Chemokine Receptor 6; CXCR4, C-X-C Motif Chemokine Receptor 4; CXCR1, C-X-C Motif Chemokine Receptor 1; CXCR2, C-X-C Motif Chemokine Receptor 2; CCR10, C-C Motif Chemokine Receptor 10; CXCR5, C-X-C Motif Chemokine Receptor 5; CCR9, C-C Motif Chemokine Receptor 9; CCR3, C-C Motif Chemokine Receptor 3; CX3CR1, C-X3-C Motif Chemokine Receptor 1; HLA-E, Major Histocompatibility Complex, Class I, E; TAP1, Transporter 1, ATP Binding Cassette Subfamily B Member; HLA-B, Major Histocompatibility Complex, Class I, B; B2M, Beta-2-Microglobulin; HLA-F, Major Histocompatibility Complex, Class I, F; HLA-DMB, Major Histocompatibility Complex, Class II, DM Beta; HLA-DOB, Major Histocompatibility Complex, Class II, DO Beta; HLA-DMA, Major Histocompatibility Complex, Class II, DM Alpha; HLA-DRB1, Major Histocompatibility Complex, Class II, DR Beta 1; HLA-DQA1, Major Histocompatibility Complex, Class II, DQ Alpha 1; HLA-DOA, Major Histocompatibility Complex, Class II, DO Alpha; HLA-DPA1, Major Histocompatibility Complex, Class II, DP Alpha 1; HLA-DPB1, Major Histocompatibility Complex, Class II, DP Beta 1; HLA-DRA, Major Histocompatibility Complex, Class II, DR Alpha; HLA-G, Major Histocompatibility Complex, Class I, G; HLA-DQA2, Major Histocompatibility Complex, Class II, DQ Alpha 2; HLA-DQB1, Major Histocompatibility Complex, Class II, DQ Beta 1; TAP2, Transporter 2, ATP Binding Cassette Subfamily B Member; TAPBP, TAP Binding Protein; HLA-A, Major Histocompatibility Complex, Class I, A; HLA-C, Major Histocompatibility Complex, Class I, C; TIGIT, T-Cell Immunoreceptor With Ig And ITIM Domains; CD96, CD96 Molecule; PDCD1, Programmed Cell Death 1; CTLA4, Cytotoxic T-Lymphocyte Associated Protein 4; BTLA, B And T Lymphocyte Associated; CD244, CD244 Molecule; PDCD1LG2, Programmed Cell Death 1 Ligand 2; HAVCR2, Hepatitis A Virus Cellular Receptor 2; LAG3, Lymphocyte Activating 3; ADORA2A, Adenosine A2a Receptor; CSF1R, Colony Stimulating Factor 1 Receptor; IL10, Interleukin 10; CD274, CD274 Molecule; IDO1, Indoleamine 2,3-Dioxygenase 1; LGALS9, Galectin 9; CD160, CD160 Molecule; KIR2DL1, Killer Cell Immunoglobulin Like Receptor, Two Ig Domains And Long Cytoplasmic Tail 1; KIR2DL3, Killer Cell Immunoglobulin Like Receptor, Two Ig Domains And Long Cytoplasmic Tail 3; NECTIN2, Nectin Cell Adhesion Molecule 2; VTCN1, V-Set Domain Containing T-Cell Activation Inhibitor 1; IL10RB, Interleukin 10 Receptor Subunit Beta; KDR, Kinase Insert Domain Receptor; TGFBR1, Transforming Growth Factor Beta 1 Transforming Growth Factor Beta Receptor 1; PVR, PVR Cell Adhesion Molecule; ULBP1, UL16 Binding Protein 1; CD276, CD276 Molecule; RAET1E, Retinoic Acid Early Transcript 1E; TNFRSF14, TNF Receptor Superfamily Member 14; TNFSF13, TNF Superfamily Member 13; TNFRSF25, TNF Receptor Superfamily Member 25; BTNL2, Butyrophilin Like 2; HHLA2, HERV-H LTR-Associating 2; IL6R, Interleukin 6 Receptor; NT5E, 5’- Nucleotidase Ecto; TNFSF9, TNF Superfamily Member 9; ICOSLG, Inducible T-Cell Costimulator Ligand; TNFSF15, TNF Superfamily Member 15; KLRK1, Killer Cell Lectin Like Receptor K1; LTA, Lymphotoxin Alpha; CD27, CD27 Molecule; CD48, CD48 Molecule; ICOS, Inducible T-Cell Costimulator; STING1, Stimulator Of Interferon Response CGAMP Interactor 1; CXCL12, C-X-C Motif Chemokine Ligand 12; MICB, MHC Class I Polypeptide-Related Sequence B; TNFSF4, TNF Superfamily Member 4; TNFRSF13C, TNF Receptor Superfamily Member 13C; IL6, Interleukin 6; TNFSF18, TNF Superfamily Member 18; ENTPD1, Ectonucleoside Triphosphate Diphosphohydrolase 1; CD40, CD40 Molecule; TNFRSF4, TNF Receptor Superfamily Member 4; CXCR4, C-X-C Motif Chemokine Receptor 4; TMIGD2, Transmembrane And Immunoglobulin Domain Containing 2; TNFRSF18, TNF Receptor Superfamily Member 18; CD70, CD70 Molecule; VSIR, V-Set Immunoregulatory Receptor; TNFRSF8, TNF Receptor Superfamily Member 8; CD28, CD28 Molecule; KLRC1, Killer Cell Lectin Like Receptor C1; CD40LG, CD40 Ligand; TNFRSF13B, TNF Receptor Superfamily Member 13B; TNFRSF17, TNF Receptor Superfamily Member 17; TNFSF14, TNF Superfamily Member 14; TNFSF13B, TNF Superfamily Member 13b; TNFRSF9, TNF Receptor Superfamily Member 9; CD80, CD80 Molecule; CD86, CD86 Molecule; IL2RA, Interleukin 2 Receptor Subunit Alpha.CCL5, C-C Motif Chemokine Ligand 5; CXCL9, C-X-C Motif Chemokine Ligand 9; CCL4, C-C Motif Chemokine Ligand 4;XCL2, X-C Motif Chemokine Ligand 2; CXCL13, C-X-C Motif Chemokine Ligand 13; CXCL10, C-X-C Motif Chemokine Ligand 10; CXCL11, C-X-C Motif Chemokine Ligand 11; XCL1, X-C Motif Chemokine Ligand 1; CCL19, C-C Motif Chemokine Ligand 19; CCL22, C-C Motif Chemokine Ligand 22; CCL18, C-C Motif Chemokine Ligand 18; CCL21, C-C Motif Chemokine Ligand 21; CCL23, C-C Motif Chemokine Ligand 23; CCL3, C-C Motif Chemokine Ligand 3; CCL8, CC Motif Chemokine Ligand 8; CCL13, C-C Motif Chemokine Ligand 13; CCL20, C-C Motif Chemokine Ligand 20; CCL24, C-C Motif Chemokine Ligand 24; CXCL16, C-X-C Motif Chemokine Ligand 16; CCL2, C-C Motif Chemokine Ligand 2; CXCL12, C-X-C Motif Chemokine Ligand 12; CCL17, C-C Motif Chemokine Ligand 17; CCL1, C-C Motif Chemokine Ligand 1; CCL7, C-C Motif Chemokine Ligand 7; CCL11, C-C Motif Chemokine Ligand 11; CCL27, C-C Motif Chemokine Ligand 27; CXCL17, C-X-C Motif Chemokine Ligand 17; CCL25, C-C Motif Chemokine Ligand 25; CX3CL1, C-X3-C Motif Chemokine Ligand 1; CCL14, C-C Motif Chemokine Ligand 14; CCL16, C-C Motif Chemokine Ligand 16; CXCL5, C-X-C Motif Chemokine Ligand 5; CXCL2, C-X-C Motif Chemokine Ligand 2; CXCL3, C-X-C Motif Chemokine Ligand 3; CCL26, C-C Motif Chemokine Ligand 26; CXCL8, C-X-C Motif Chemokine Ligand 8; CXCL1, C-X-C Motif Chemokine Ligand 1; CXCL6, C-X-C Motif Chemokine Ligand 6; CCL15, C-C Motif Chemokine Ligand 15; CCL28, C-C Motif Chemokine Ligand 28; CXCL14, C-X-C Motif Chemokine Ligand 14; XCR1, X-C Motif Chemokine Receptor 1; CCR1, C-C Motif Chemokine Receptor 1; CCR2, C-C Motif Chemokine Receptor 2; CCR8, C-C Motif Chemokine Receptor 8; CCR4, C-C Motif Chemokine Receptor 4; CCR7, C-C Motif Chemokine Receptor 7; CXCR3, C-X-C Motif Chemokine Receptor 3; CCR5, C-C Motif Chemokine Receptor 5; CXCR6, C-X-C Motif Chemokine Receptor 6; CCR6, C-C Motif Chemokine Receptor 6; CXCR4, C-X-C Motif Chemokine Receptor 4; CXCR1, C-X-C Motif Chemokine Receptor 1; CXCR2, C-X-C Motif Chemokine Receptor 2; CCR10, C-C Motif Chemokine Receptor 10; CXCR5, C-X-C Motif Chemokine Receptor 5; CCR9, C-C Motif Chemokine Receptor 9; CCR3, C-C Motif Chemokine Receptor 3; CX3CR1, C-X3-C Motif Chemokine Receptor 1; HLA-E, Major Histocompatibility Complex, Class I, E; TAP1, Transporter 1, ATP Binding Cassette Subfamily B Member; HLA-B, Major Histocompatibility Complex, Class I, B; B2M, Beta-2-Microglobulin; HLA-F, Major Histocompatibility Complex, Class I, F; HLA-DMB, Major Histocompatibility Complex, Class II, DM Beta; HLA-DOB, Major Histocompatibility Complex, Class II, DO Beta; HLA-DMA, Major Histocompatibility Complex, Class II, DM Alpha; HLA-DRB1, Major Histocompatibility Complex, Class II, DR Beta 1; HLA-DQA1, Major Histocompatibility Complex, Class II, DQ Alpha 1; HLA-DOA, Major Histocompatibility Complex, Class II, DO Alpha; HLA-DPA1, Major Histocompatibility Complex, Class II, DP Alpha 1; HLA-DPB1, Major Histocompatibility Complex, Class II, DP Beta 1; HLA-DRA, Major Histocompatibility Complex, Class II, DR Alpha; HLA-G, Major Histocompatibility Complex, Class I, G; HLA-DQA2, Major Histocompatibility Complex, Class II, DQ Alpha 2; HLA-DQB1, Major Histocompatibility Complex, Class II, DQ Beta 1; TAP2, Transporter 2, ATP Binding Cassette Subfamily B Member; TAPBP, TAP Binding Protein; HLA-A, Major Histocompatibility Complex, Class I, A; HLA-C, Major Histocompatibility Complex, Class I, C; TIGIT, T-Cell Immunoreceptor With Ig And ITIM Domains; CD96, CD96 Molecule; PDCD1, Programmed Cell Death 1; CTLA4, Cytotoxic T-Lymphocyte Associated Protein 4; BTLA, B And T Lymphocyte Associated; CD244, CD244 Molecule; PDCD1LG2, Programmed Cell Death 1 Ligand 2; HAVCR2, Hepatitis A Virus Cellular Receptor 2; LAG3, Lymphocyte Activating 3; ADORA2A, Adenosine A2a Receptor; CSF1R, Colony Stimulating Factor 1 Receptor; IL10, Interleukin 10; CD274, CD274 Molecule; IDO1, Indoleamine 2,3-Dioxygenase 1; LGALS9, Galectin 9; CD160, CD160 Molecule; KIR2DL1, Killer Cell Immunoglobulin Like Receptor, Two Ig Domains And Long Cytoplasmic Tail 1; KIR2DL3, Killer Cell Immunoglobulin Like Receptor, Two Ig Domains And Long Cytoplasmic Tail 3; NECTIN2, Nectin Cell Adhesion Molecule 2; VTCN1, V-Set Domain Containing T-Cell Activation Inhibitor 1; IL10RB, Interleukin 10 Receptor Subunit Beta; KDR, Kinase Insert Domain Receptor; TGFBR1, Transforming Growth Factor Beta 1 Transforming Growth Factor Beta Receptor 1; PVR, PVR Cell Adhesion Molecule; ULBP1, UL16 Binding Protein 1; CD276, CD276 Molecule; RAET1E, Retinoic Acid Early Transcript 1E; TNFRSF14, TNF Receptor Superfamily Member 14; TNFSF13, TNF Superfamily Member 13; TNFRSF25, TNF Receptor Superfamily Member 25; BTNL2, Butyrophilin Like 2; HHLA2, HERV-H LTR-Associating 2; IL6R, Interleukin 6 Receptor; NT5E, 5’- Nucleotidase Ecto; TNFSF9, TNF Superfamily Member 9; ICOSLG, Inducible T-Cell Costimulator Ligand; TNFSF15, TNF Superfamily Member 15; KLRK1, Killer Cell Lectin Like Receptor K1; LTA, Lymphotoxin Alpha; CD27, CD27 Molecule; CD48, CD48 Molecule; ICOS, Inducible T-Cell Costimulator; STING1, Stimulator Of Interferon Response CGAMP Interactor 1; CXCL12, C-X-C Motif Chemokine Ligand 12; MICB, MHC Class I Polypeptide-Related Sequence B; TNFSF4, TNF Superfamily Member 4; TNFRSF13C, TNF Receptor Superfamily Member 13C; IL6, Interleukin 6; TNFSF18, TNF Superfamily Member 18; ENTPD1, Ectonucleoside Triphosphate Diphosphohydrolase 1; CD40, CD40 Molecule; TNFRSF4, TNF Receptor Superfamily Member 4; CXCR4, C-X-C Motif Chemokine Receptor 4; TMIGD2, Transmembrane And Immunoglobulin Domain Containing 2; TNFRSF18, TNF Receptor Superfamily Member 18; CD70, CD70 Molecule; VSIR, V-Set Immunoregulatory Receptor; TNFRSF8, TNF Receptor Superfamily Member 8; CD28, CD28 Molecule; KLRC1, Killer Cell Lectin Like Receptor C1; CD40LG, CD40 Ligand; TNFRSF13B, TNF Receptor Superfamily Member 13B; TNFRSF17, TNF Receptor Superfamily Member 17; TNFSF14, TNF Superfamily Member 14; TNFSF13B, TNF Superfamily Member 13b; TNFRSF9, TNF Receptor Superfamily Member 9; CD80, CD80 Molecule; CD86, CD86 Molecule; IL2RA, Interleukin 2 Receptor Subunit Alpha.

The results showed that TIGIT exhibited a significant coexpression relationship with most chemokines, receptors, major histocompatibility complex (MHC), immunoinhibitors and immunostimulators. Notably, TIGIT expression was positively correlated with the expression of programmed cell death 1 (PDCD1), programmed cell death 1 ligand 2 (PDCD1LG2), cytotoxic T-lymphocyte associated protein 4 (CTLA4), lymphocyte activating 3 (LAG3), indoleamine 2,3-dioxygenase 1 (IDO1), interleukin 10 (IL-10), and transforming growth factor beta 1 (TGFB1), especially in gastroesophageal tumors and melanoma (**Figure 11**).

The correlation coefficients are presented in detail in **Supplementary Table 9**.

#### 3.2.6 Associations of TIGIT expression with tumor mutational burden (TMB) and microsatellite instability (MSI)

**Figure 12:**
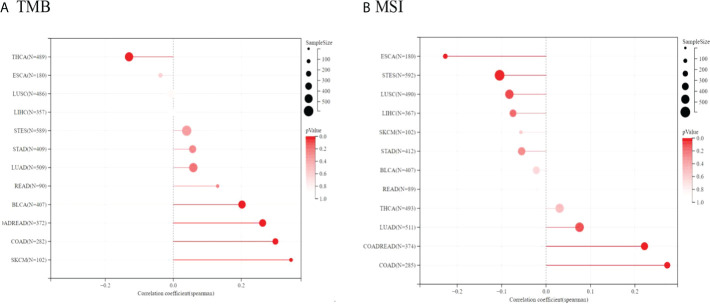
Spearman correlation between TMB, MSI and TIGIT gene expression were presented in **(A, B)** respectively. The correlation coefficient is presented by using the length of the lollipop. The redder the color, the more statistically significant it is.

Because of the essential roles of TMB and MSI in the prediction of the response to immune therapy, Spearman correlation analysis was conducted to assess the relationship between TIGIT expression, TMB and MSI. The results showed that TIGIT expression was positively related to TMB in BLCA, COADREAD, COAD, and SKCM but negatively related to TMB in THCA. Moreover, TIGIT expression was positively correlated with MSI in COAD, COADREAD, and LUAD, while it was negatively correlated with MSI in ESCA and STES (see **Figure 12**). The correlation coefficients and P values are presented in **Supplementary Tables 10, 11**.

The results strongly indicated that TIGIT was well associated with tumor immunity. Therefore, TIGIT might be considered a promising biomarker for predicting the immunotherapy response.

#### 3.2.7 Construction of the protein protein interaction (PPI) network, gene ontology (GO) and Kyoto encyclopedia of gene and genomes (KEGG) enrichment analysis

**Figure 13:**
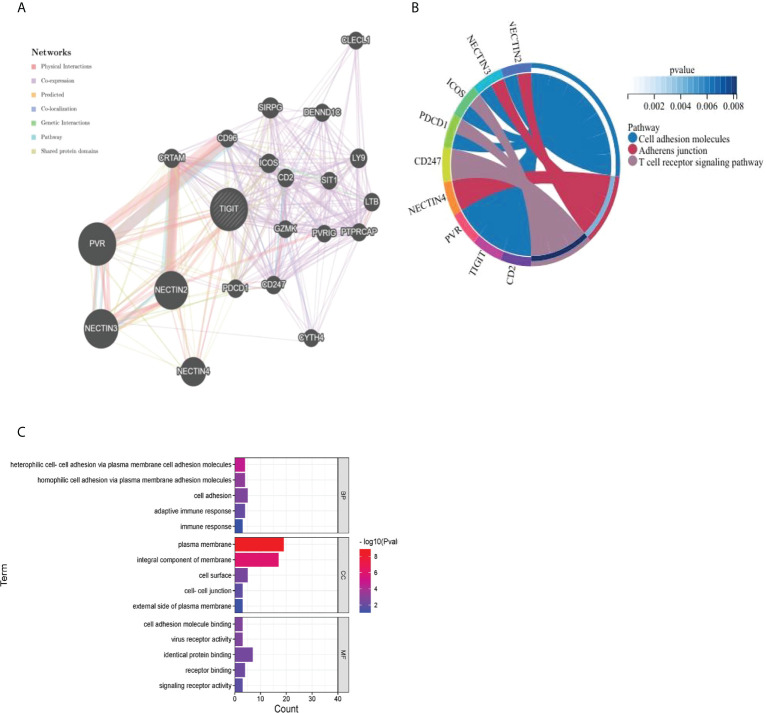
Visualization and enrichment analysis for genes that interacted with TIGIT **(A)** PPI network **(B)** Chord diagram for KEGG analysis **(C)** Bar graph for GO analysis).

As presented in **Figure 13A**, we utilized the GeneMANIA online program to create a PPI network for 21 genes that interacted with TIGIT. TIGIT was found to interact with PDCD1, which indicated that patients with resistance to PD-1 inhibitors might benefit from the combination of TIGIT inhibitors. The biological processes (BP) enriched in this gene set were primarily those related to cell adhesion, heterophilic cell−cell adhesion *via* plasma membrane cell adhesion molecules and homophilic cell adhesion *via* plasma membrane adhesion molecules, while the cellular components (CC) enriched were plasma membrane AND integral component of membrane. The enriched molecular functions (MF) were linked to identical protein binding and receptor binding. The KEGG results showed that they were mainly enriched in the cell adhesion molecules, adherens junction and T-cell receptor signaling pathways (**Figures 13B, C**).

#### 3.2.8 Construction of the ceRNA regulatory network

**Figure 14:**
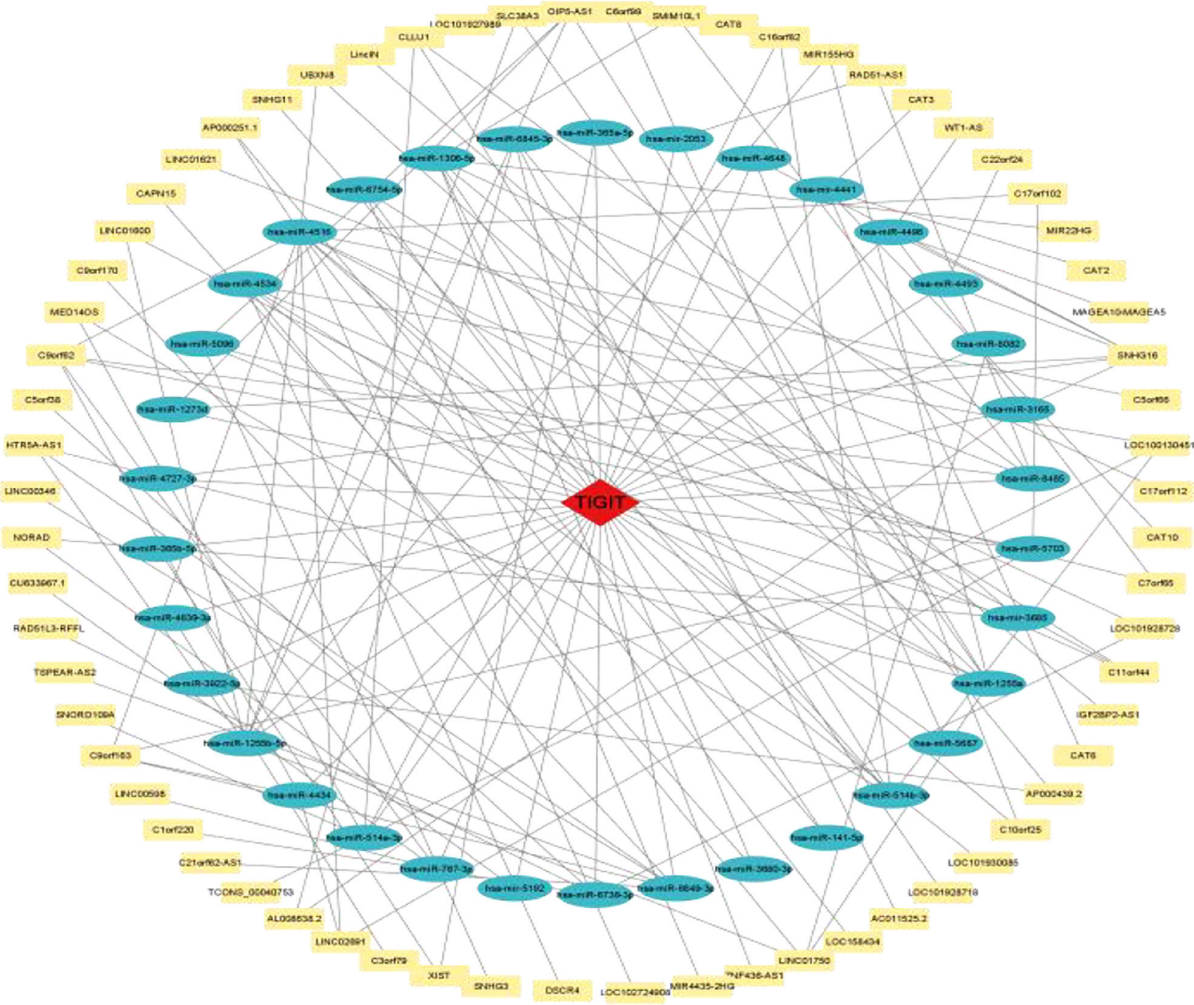
TIGIT-miRNA−lncRNA network.

As shown in **Figure 14**, a ceRNA coexpression network consisting of 70 lncRNAs, 34 miRNAs, and 1 mRNA was visualized by Cytoscape after merging these predicted results. By using the CytoHubba plug-in in Cytoscape, we screened out the top 10 node degrees to represent the central genes of the PPI network, including TIGIT, hsa-miR-4516, hsa-miR-1255a, hsa-miR-1255b-5p, hsa-miR-1306-5p, hsa-miR-514a-3p, hsa-miR-6849-3p, hsa-miR-514b-3p, SNHG16, and hsa-miR-4534.

## 4 Discussion

The results of the meta-analysis showed that high expression of TIGIT was associated with poorer OS, PFS, RFS and DFS in East Asian patients with solid cancers. In contrast to the study reported by Kunmin Xiao et al (42), we also discussed the relationship between TIGIT and DFS and RFS. DFS and RFS are important clinical outcomes for cancers with relatively good clinical prognosis. Most importantly, we discussed the heterogeneity caused by postoperative treatments, which might have a very important impact on the prognosis of cancer patients. It was found in our study that the cancer type, sample sizes, and different cutoff values might be the source of heterogeneity. The high expression of TIGIT was not significantly correlated with poor PFS of PSCCE, RFS of MTC, DFS of PTC or OS of SCLC, CRC, MTC, ATC, PDTC and PTC. Studies with sample sizes <100 did not support the relationship between high expression of TIGIT and OS or DFS, while studies taking CPS≥1 as the cutoff value did not support the relationship between high expression of TIGIT and OS or RFS. Whether postoperative treatments result in heterogeneity still needs further study.

SCLC is a classical neuroendocrine tumor with low immunogenicity and low MHC I expression levels, which makes it difficult to recognize by CD8 T-cell receptors. Its immune regulation is more complex than that of other solid tumors due to the existence of autocrine or paracrine molecules. In ATC, TIGIT expression is not found to have prognostic value (38). The reason may be that ATC patients suffer from extremely short survival, and the sample size may be too small to distinguish the prognosis.

In our meta-analysis, a relationship between high expression of TIGIT and poorer OS was not found in the studies taking CPS≥1 as the cutoff value. The tumor proportion score (TPS) and the combined positive score (CPS) have been widely used in clinical research. TPS calculates the ratio of TIGIT-stained tumor cells to the total number of viable tumor cells, while CPS calculates the ratio of potential TIGIT expression, including tumor cells and immune cells, to the total number of viable tumor cells. However, it remains unclear which is more suitable for assessing TIGIT expression as a prognostic biomarker (43).

Vascular endothelial cells, nonmalignant cells, immunocytes, tumor-associated macrophages (TAMs), cancer-associated fibroblasts (CAFs), myeloid-derived suppressor cells (MDSCs), natural killer (NK) cells, dendritic cells (DCs) and tumor-associated neutrophils (TANs) make up the tumor microenvironment (TME) (44, 45). TIGIT was highly positively correlated with the ESTIMATE score in all of the cancers included, which indicated an advanced cancer stage with a poor prognosis.

Tumor-infiltrating immunocytes can promote or antagonize tumorigenesis and progression (46). TIGIT expressed on TILs responded to the TME. TIGIT marks the most dysfunctional subset of CD8+ T cells and Tregs with a highly suppressive function (47). In this study, high TIGIT expression promoted the infiltration levels of CD8 T cells, M1 macrophages, naive B cells, activated memory CD4 T cells, Tregs and activated NK cells while inhibiting the infiltration levels of activated dendritic cells and mast cells in most of the cancers included. Among them, M1 macrophages, activated memory CD4 T cells, activated dendritic cells, mast cells and activated NK cells play antitumor roles in the TME and are related to better outcomes, while Tregs play immune suppression roles and are related to worse survival prognosis (48). A positive correlation was discovered between the expression of TIGIT and most of the other immune checkpoints, especially in ESCA, STAD, STES and SKCM (12, 47, 49, 50). This result suggested that TIGIT might be involved in different immune responses and immunocyte infiltration. The combined blockade of TIGIT and other new immune checkpoints may be a possible option for immunotherapy, especially in patients with gastroesophageal tumors and melanoma.

MSI and TMB are two valuable indexes suggesting the sensitivity of immune checkpoint inhibitors. TMB can induce new antigens to facilitate immune recognition. MSI caused by hypermutability (gain or loss) of nucleotides from DNA elements is associated with increased expression of neoantigens, higher PD-L1 expression and TMB-H (51). In this study, TIGIT expression was positively related to TMB and MSI in COAD and COADREAD, which indicated that patients with COAD or COADREAD might benefit from TIGIT inhibitors.

Regarding the possible regulatory mechanisms, the results of the GO and KEGG enrichment analyses indicated that TIGIT was closely related to the functions of cell adhesion, adherens junction and the T-cell receptor signaling pathway, which supported the oncogenic role and immunological function of TIGIT in the tumor immune microenvironment.

Currently, it is urgent to find new immune checkpoints to compensate for drug resistance and severe adverse reactions caused by PD-1/PD-L1 and CTLA4 inhibitors. Studies on TIGIT expression provided more encouraging results than those on LAG-3 and TIM-3 (52). TIGIT inhibitors, such as tibolumab, vibostolimab, ocperlimab, M-6223, ASP-8374, COM-902 and IBI-939, have been under clinical trials in patients with non-small cell lung cancer (NSCLC), esophageal squamous cell carcinoma (ESCC) and gastric adenocarcinoma (GAC). TIGIT expression paralleled that of PD-1 (2). Most TIGIT inhibitors are used in combination with PD-L1/PD-1 inhibitors, such as zimberelimab and atezolizumab (53, 54). We will continue to follow up the results of relevant clinical reports.

There were some limitations in our meta-analysis. Over half of the studies did not report postoperative therapy, which led to some bias in our analysis. Second, all of the subjects were Asian, and whether the conclusion could be applied to other populations remained uncertain. Third, the scale of the included studies was limited. Some parts of the subgroup analysis only included one kind of cancer. Large sample size studies are still needed to determine the relationship between TIGIT expression and survival prognosis, especially PFS, RFS and DFS.

## 5 Conclusion

TIGIT is valuable in predicting the survival prognosis of patients with solid cancers. TIGIT is correlated with the TME, infiltration of immune cells, immune-related genes, MSI and TMB. The results indicate the role of TIGIT in tumoriFabbrevgenesis and progression. TIGIT inhibitors may be promising choices for solid cancers in the future.

## Data availability statement

The original contributions presented in the study are included in the article/**Supplementary Material**. Further inquiries can be directed to the corresponding author.

## Author contributions

SL and LL contributed equally to article review, manuscript writing and revisions for intellectual content. TP and XL were responsible for data extraction and English translation. YT was responsible for data extraction, English translation and quality assessment. YJ was the corresponding author and was responsible for quality assessment and revisions for intellectual content. All authors contributed to the article and approved the submitted version.

## Acknowledgments

The authors would like to thank all the scholars who made constant efforts for cancer research.

## Conflict of interest

The authors declare that the research was conducted in the absence of any commercial or financial relationships that could be construed as a potential conflict of interest.

## Publisher’s note

All claims expressed in this article are solely those of the authors and do not necessarily represent those of their affiliated organizations, or those of the publisher, the editors and the reviewers. Any product that may be evaluated in this article, or claim that may be made by its manufacturer, is not guaranteed or endorsed by the publisher.

## Glossary

**Table:**

	
Bladder Urothelial Carcinoma	BLCA
Colon adenocarcinoma	COAD
Colon adenocarcinoma/Rectum adenocarcinoma Esophageal carcinoma	COADREAD
Esophageal carcinoma	ESCA
Liver hepatocellular carcinoma	LIHC
Lung squamous cell carcinoma	LUSC
Rectum adenocarcinoma	READ
Skin Cutaneous Melanoma	SKCM
Stomach adenocarcinoma	STAD
Stomach and Esophageal carcinoma	STES
activated dendritic cells	DCs
advanced thyroid carcinoma	ATC
anaplastic thyroid carcinoma	ATC
bladder urothelial carcinoma	BLCA
	
cancer-associated fibroblasts	CAFs
cancer-associated fibroblasts	CAFs
China national knowledge infrastructure	CNKI
colon adenocarcinoma	COAD
colon adenocarcinoma/Rectum adenocarcinoma esophageal carcinoma	COADREAD
colorectal cancer	CRC
combined positive score	CPS
cytotoxic T-lymphocyte associated protein 4	CTLA4
defective DNA mismatch repair	dMMR
dendritic cells	DCs
disease free survival	DFS
disease-free survival	DFS
esophageal carcinoma	ESCA
esophageal squamous cell carcinoma	ESCC
follicular helper T cells	Tfh
gastric adenocarcinoma	GAC
gastric cancer	GC
Hepatitis B virus hepatocellular carcinoma	HBV-HCC
indoleamine 2 3-dioxygenase 1	IDO1
Interleukin 10	IL-10
liver hepatocellular carcinoma	LIHC
locally advanced papillary thyroid carcinoma	PTC
lung adenocarcinoma	LUAD
lung squamous cell carcinoma	LUSC
lymphocyte activating 3	LAG3
Microsatellite instability	MSI
muscle-invasive bladder cancer	MIBC
muscle-invasive bladder cancer	MIBC
myeloid-derived suppressor cells	MDSCs
myeloid-derived suppressor cells	MDSCs
	
natural killer	NK
Newcastle−Ottawa Quality Assessment Scale	NOS
non-small cell lung cancer	NSCLC
overall survival	OS
poorly differentiated thyroid carcinoma	PDTC
Preferred Reporting Items for Systematic Reviews and Meta-Analyses	PRISMA
primary small cell carcinoma of esophagus	PSCCE
programmed cell death 1 ligand 2	PDCD1LG2
programmed cell death 1	PDCD1
progression-free survival	PFS
Randomized controlled Trial	RCT
Rectum adenocarcinoma	READ
recurrence free survival	RFS
regulatory T cells	Tregs
skin cutaneous melanoma	SKCM
small cell lung cancer	SCLC
stomach adenocarcinoma	STAD
stomach and esophageal carcinoma	STES
structural recurrence free survival	SRFS
Thyroid carcinoma	THCA
transforming Growth Factor Beta 1	TGFB1
tumor infiltrating lymphocytes	TILs
tumor microenvironment	TME
tumor proportion score	TPS
tumor-associated macrophages	TAMs
tumor-associated neutrophils	TANs
tumor-associated neutrophils	TANs
V-set and immunoglobulin domain-containing protein 9	VSIG9
V-set and transmembrane do-main-containing protein 3	VSTM3
Washington University cell adhesion molecule	WUCAM
C-C Motif Chemokine Ligand 5	CCL5
C-X-C Motif Chemokine Ligand 9	CXCL9
C-C Motif Chemokine Ligand 4	CCL4
X-C Motif Chemokine Ligand 2	XCL2
C-X-C Motif Chemokine Ligand 13	CXCL13
C-X-C Motif Chemokine Ligand 10	CXCL10
C-X-C Motif Chemokine Ligand 11	CXCL11
X-C Motif Chemokine Ligand 1	XCL1
C-C Motif Chemokine Ligand 19	CCL19
CC Motif Chemokine Ligand 22	CCL22
C-C Motif Chemokine Ligand 18	CCL18
C-C Motif Chemokine Ligand 21	CCL21
C-C Motif Chemokine Ligand 23	CCL23
C-C Motif Chemokine Ligand 3	CCL3
C-C Motif Chemokine Ligand 8	CCL8
C-C Motif Chemokine Ligand 13	CCL13
CC Motif Chemokine Ligand 20	CCL20
C-C Motif Chemokine Ligand 24	CCL24
	
C-X-C Motif Chemokine Ligand 16	CXCL16
C-C Motif Chemokine Ligand 2	CCL2
C-X-C Motif Chemokine Ligand 12	CXCL12
C-C Motif Chemokine Ligand 17	CCL17
C-C Motif Chemokine Ligand 1	CCL1
C-C Motif Chemokine Ligand 7	CCL7
C-C Motif Chemokine Ligand 11	CCL11
C-C Motif Chemokine Ligand 27	CCL27
C-X-C Motif Chemokine Ligand 17	CXCL17
C-C Motif Chemokine Ligand 25	CCL25
C-X3-C Motif Chemokine Ligand 1	CX3CL1
C-C Motif Chemokine Ligand 14	CCL14
C-C Motif Chemokine Ligand 16	CCL16
C-X-C Motif Chemokine Ligand 5	CXCL5
C-X-C Motif Chemokine Ligand 2	CXCL2
C-X-C Motif Chemokine Ligand 3	CXCL3
C-C Motif Chemokine Ligand 26	CCL26
C-X-C Motif Chemokine Ligand 8	CXCL8
C-X-C Motif Chemokine Ligand1	CXCL1
C-X-C Motif Chemokine Ligand 6	CXCL6
C-C Motif Chemokine Ligand 15	CCL15
C-C Motif Chemokine Ligand 28	CCL28
C-X-C Motif Chemokine Ligand 14	CXCL14
X-C Motif Chemokine Receptor 1	XCR1
C-C Motif Chemokine Receptor 1	CCR1
C-C Motif Chemokine Receptor 2	CCR2
C-C Motif Chemokine Receptor 8	CCR8
CCMotif Chemokine Receptor 4	CCR4
C-C Motif Chemokine Receptor 7	CCR7
C-X-C Motif Chemokine Receptor 3	CXCR3
C-C Motif Chemokine Receptor 5	CCR5
C-X-C Motif Chemokine Receptor 6	CXCR6
C-C Motif Chemokine Receptor 6	CCR6
C-X-C MotifChemokine Receptor 4	CXCR4
C-X-C Motif Chemokine Receptor 1	CXCR1
C-X-C Motif Chemokine Receptor 2	CXCR2
C-C Motif Chemokine Receptor 10	CCR10
C-X-C Motif Chemokine Receptor 5	CXCR5
C-C Motif Chemokine Receptor 9	CCR9
C-C Motif Chemokine Receptor 3	CCR3
C-X3-C Motif Chemokine Receptor 1	CX3CR1
Major Histocompatibility Complex Class I E	HLA-E
Transporter 1 ATP Binding Cassette Subfamily B Member	TAP1
Major Histocompatibility Complex Class I B	HLA-B
Beta-2-Microglobulin	B2 M
Major Histocompatibility Complex Class I F	HLA-F
Major Histocompatibility Complex Class II DM Beta	HLA-DMB
Major Histocompatibility Complex Class II DO Beta	HLA-DOB
Major Histocompatibility Complex Class II DM Alpha	HLA-DMA
	
Major Histocompatibility Complex Class II DR Beta 1	HLA-DRB1
Major Histocompatibility Complex Class II DQ Alpha 1	HLA-DQA1
Major Histocompatibility Complex Class II DO Alpha	HLA-DOA
Major Histocompatibility Complex Class II DP Alpha 1	HLA-DPA1
Major Histocompatibility Complex Class II DP Beta 1	HLA-DPB1
Major Histocompatibility Complex Class II DR Alpha	HLA-DRA
Major Histocompatibility Complex Class I G	HLA-G
Major Histocompatibility Complex Class II DQ Alpha 2	HLA-DQA2
Major Histocompatibility Complex Class II DQ Beta 1	HLA-DQB1
Transporter 2 ATP Binding Cassette Subfamily B Member	TAP2
TAP Binding Protein	TAPBP
Major Histocompatibility Complex Class I A	HLA-A
Major Histocompatibility Complex Class I C	HLA-C
T-Cell Immunoreceptor With Ig And ITIM Domains	TIGIT
CD96 Molecule	CD96
Programmed Cell Death 1	PDCD1
Cytotoxic T-Lymphocyte Associated Protein 4	CTLA4
B And T Lymphocyte Associated	BTLA
CD244 Molecule	CD244
Programmed Cell Death 1 Ligand 2	PDCD1LG2
Hepatitis A Virus Cellular Receptor 2	HAVCR2
Lymphocyte Activating 3	LAG3
Adenosine A2a Receptor	ADORA2A
Colony Stimulating Factor 1 Receptor	CSF1R
Interleukin 10	IL10
CD274 Molecule	CD274
Indoleamine 2, 3-Dioxygenase 1	IDO1
Galectin 9	LGALS9
CD160 Molecule	CD160
Killer Cell Immunoglobulin	
