# Supplementary material for: Prognostic value of TIGIT in East Asian patients with solid cancers: A systematic review, meta-analysis and pancancer analysis
Source: Front Immunol. 2022 Sep 21;13:977016. doi: 10.3389/fimmu.2022.977016 (PMC9532506; doi:10.3389/fimmu.2022.977016)
Supplement: Supplementary file 1 [file DataSheet_1.pdf]

Supplementary materials

Table 1 English literature retrieval strategy(Pubmed)

Number	Search terms
1	TIGIT [Title/Abstract]
2	T-cell Ig and ITIM domain [Title/Abstract]
3	Vstm3[Title/Abstract]
4	WUCAM [Title/Abstract]
5	VSIG9[Title/Abstract]
6	1 OR 2 OR 3 OR 4 OR 5
7	Carcinoma [Title/Abstract]
8	Carcinoma[MeSH]
9	Neoplasms[Title/Abstract]
10	Neoplasms[MeSH]
11	cancer [Title/Abstract]
12	neoplasia [Title/Abstract]
13	Tumor [Title/Abstract]
14	malignancy [Title/Abstract]
15	malignant neoplasm (Full text)
16	7 OR 8 OR 9 OR 10 OR 11 OR 12 OR 13 OR 14 OR 15
17	Prognosis [MeSH]
18	Prognosis [Title/Abstract]
19	Survival Analysis [MeSH]
20	Survival Analysis [Title/Abstract]
21	Prognostic [Title/Abstract]
22	Treatment Outcome [MeSH]
23	Treatment Outcome [Title/Abstract]
24	Fatal Outcome [MeSH]
25	Fatal Outcome [Title/Abstract]
26	Mortality [MeSH]
27	Mortality [Title/Abstract]
28	17 OR 18 OR 19 OR 20 OR 21 OR 22 OR 23 OR 24 OR 25 OR 26 OR 27
29	6 AND 16 AND 28

Table 2 literature search strategy(Embase)

Number	Search terms
1	TIGIT [Title/Abstract]
2	T-cell Ig and ITIM domain [Title/Abstract]
3	Vstm3[Title/Abstract]

4	WUCAM [Title/Abstract]
5	VSIG9[Title/Abstract]
6	1 OR 2 OR 3 OR 4 OR 5
7	Carcinoma [Title/Abstract]
8	Neoplasms[Title/Abstract]
9	cancer [Title/Abstract]
10	neoplasia [Title/Abstract]
11	Tumor [Title/Abstract]
12	malignancy [Title/Abstract]
13	malignant neoplasm [Title/Abstract]
14	7 OR 8 OR 9 OR 10 OR 11 OR 12 OR 13
15	Prognos* [Title/Abstract]
16	Survival Analysis [Title/Abstract]
17	Treatment Outcome [Title/Abstract]
18	Fatal Outcome [Title/Abstract]
19	Mortality [Title/Abstract]
20	15 OR 16 OR 17 OR 18 OR 19
21	6 AND 14 AND 20

Table 3 Literature search strategy(Cochrane library)

Number	Search terms
1	TIGIT [Title Abstract Keyword]
2	T-cell Ig and ITIM domain [Title Abstract Keyword]
3	Vstm3[Title Abstract Keyword]
4	WUCAM [Title Abstract Keyword]
5	VSIG9[Title Abstract Keyword]
6	1 OR 2 OR 3 OR 4 OR 5
7	Carcinoma [Title Abstract Keyword]
8	Neoplasms[Title Abstract Keyword]
9	cancer [Title Abstract Keyword]
10	neoplasia [Title Abstract Keyword]
11	Tumor [Title Abstract Keyword]
12	malignancy [Title Abstract Keyword]
13	malignant neoplasm [Title Abstract Keyword]
14	7 OR 8 OR 9 OR 10 OR 11 OR 12 OR 13
15	Prognos* [Title Abstract Keyword]
16	Survival Analysis [Title Abstract Keyword]
17	Treatment Outcome [Title Abstract Keyword]
18	Fatal Outcome [Title Abstract Keyword]
19	Mortality [Title Abstract Keyword]
20	15 OR 16 OR 17 OR 18 OR 19

21	6 AND 14 AND 20

Table 4 Quality assessment of included studies according to the Newcastle-Ottawa Scale.

**Study**	**Selection**				**Comparability**		**Outcome**			**In total**
	1*	2*	3*	4*	5*	6*	7*	8*	9*	
Zhao JJ 2018	1	1	1	1	1	0	1	1	1	8
Tang W 2019	1	1	1	1	1	0	1	1	1	8
Xu Y 2019	1	1	1	1	1	0	1	0	0	6
Lee WJ 2020	1	1	1	1	1	0	1	0	1	7
Sun Y 2020	1	1	1	1	1	0	1	0	1	7
Zhao K 2020	1	1	1	1	1	0	1	1	1	8
Zhou X 2020	1	1	1	1	1	0	1	1	1	8
Liang R 2021	1	1	1	1	1	0	1	0	0	6
Liu HF 2022	1	1	1	1	1	0	1	1	0	7
Wang PP 2021	1	1	1	1	1	0	1	1	1	8
Daisuke Murakami 2022	1	1	1	1	1	0	1	0	1	7
Jiang C 2022	1	1	1	1	1	0	1	1	1	8
Yu LH 2021	1	1	1	1	1	0	1	1	1	8
Shi X 2021	1	1	1	1	1	0	1	1	1	8
Liu ZP 2020	1	1	1	1	1	0	1	1	1	8
Luo Y 2022	1	1	1	1	1	0	1	1	0	7

1*: Representativeness of the exposed cohort, 2*: Selection of the non exposed cohort, 3*: Ascertainment of exposure, 4*: Demonstration that outcome of interest was not present at start of study, 5*: Study controls for the most important factor, 6*: Study controls for any additional factor, 7*: Assessment of outcome, 8*: Was follow-up long enough for outcomes to occur, 9*: Adequacy of follow up of cohorts.

Table 5 Differential TIGIT mRNA expression in 12 kinds of cancers and normal tissues

Cancer type	Tumor	Normal	P value
LUAD	1.44±1.33	0.46±1.12	p=1.5e-32
ESCA	0.29±1.59	-2.88±2.23	p=3.7e-59
STES	0.71±1.57	-2.60±2.18	p=4.8e-156
COADREAD	-0.03±1.60	-1.93±2.39	p=4.2e-29
LIHC	-1.18±1.86	-1.71±1.23	p=3.4e-3
SKCM	-0.10±2.07	-2.56±1.70	p=3.7e-27
THCA	-0.53±2.05	-0.95±2.03	p=1.2e-4
COAD	0.01±1.62	-2.00±2.38	p=4.7e-28
STAD	0.89±1.53	-1.74±1.76	p=1.8e-54
READ	-0.16±1.55	0.46±1.04	P=0.11
LUSC	1.25±1.36	0.46±1.12	p=1.1e-22
BLCA	0.12±1.98	-0.89±3.04	P=0.09

Table 6 TIGIT expression in patients with wild type or SNV

Cancer type	Wild type	Mutation type	P value
LUAD	1.44±1.32	1.55±1.50	0.75
COAD	8.1e-3±1.63	-0.13±1.97	0.91
COADREAD	-0.04±1.62	-0.10±1.54	0.92
BRCA	0.64±1.93	2.27±1.27	0.11
STES	0.69±1.57	1.24±1.82	0.59
STAD	0.87±1.52	1.92±1.50	0.27

Table 7 TIGIT expression in patients with with or without CNV

Cancer type	Observation group(Mean±std)	control group(Mean±std)	P value for wilcoxon rank sum test	P value for Kruskal-Wallis rank sum test
LUAD(Gain=20,Neutral=481,Loss=10)	Gain(1.28±1.24)	Neutral(1.45±1.33)	0.55	0.76
	Gain(1.28±1.24)	Loss(1.34±1.29)	0.91	
	Neutral(1.45±1.33)	Loss(1.34±1.29)	0.66	
COADREAD(Neutral=372,Loss=3)	Neutral(-0.03±1.61)	Loss(-0.59±1.43)	0.43	0.43
BRCA(Neutral=1004, Gain=52,Loss=27)	Neutral(0.60±1.93)	Gain(0.97±1.84)	0.07	0.2
	Neutral(0.60±1.93)	Loss(0.49±2.10)	0.94	
	Gain(0.97±1.84)	Loss(0.49±2.10)	0.24	
ESCA(Neutral=160,Loss=6,Gain=14)	Neutral(0.31±1.60)	Loss(-0.20±1.35)	0.38	0.62
	Neutral(0.31±1.60)	Gain(0.24±1.64)	0.64	
	Loss(-0.20±1.35)	Gain(0.24±1.64)	0.84	
STES(Neutral=552,Loss=16,Gain=23)	Neutral(0.73±1.57)	Loss(0.55±1.42)	0.61	0.09
	Neutral(0.73±1.57)	Gain(0.16±1.59)	0.03	
	Loss(0.55±1.42)	Gain(0.16±1.59)	0.25	
STAD(Neutral=392,Gain=9,Loss=10)	Neutral(0.90±1.53)	Gain(0.05±1.60)	0.04	0.11
	Neutral(0.90±1.53)	Loss(1.00±1.32)	0.88	
	Gain(0.05±1.60)	Loss(1.00±1.32)	0.08	
LUSC(Neutral=415,Lo	Neutral(1.34±1.33)	Loss(1.05±1.50)	0.24	4.40e-03

ss=38,Gain=44)	Neutral(1.34±1.33)	Gain(0.62±1.34)	1.50E-03	
	Loss(1.05±1.50)	Gain(0.62±1.34)	0.26	
LIHC(Neutral=357,Gain=5,Loss=5)	Neutral(-1.21±1.85)	Gain(-0.33±2.40)	0.45	0.1
	Neutral(-1.21±1.85)	Loss(0.41±1.40)	0.04	
	Gain(-0.33±2.40)	Loss(0.41±1.40)	0.69	
SKCM(Neutral=95,Loss=6)	Neutral(-0.19±2.05)	Loss(1.67±1.64)	0.03	0.03
BLCA(Neutral=375,Loss=12,Gain=18)	Neutral(0.10±1.99)	Loss(0.39±1.82)	0.57	0.77
	Neutral(0.10±1.99)	Gain(0.19±1.92)	0.64	
	Loss(0.39±1.82)	Gain(0.19±1.92)	0.95	

Table 8 Correlation coefficient between TIGIT expression and immune cell infiltration (CIBERSORT)

Immune cell	LUAD	ESCA	STES	COAD	COADREAD	STAD	LUSC	LIHC	SKCM	BLCA	THCA	READ
B_cells_naive	-0.019	0.300	0.231	0.187	0.202	0.152	0.062	0.191	0.210	0.361	0.532	0.259
B_cells_memory	0.148	-0.069	0.066	-0.056	-0.084	0.118	0.047	-0.062	0.063	-0.361	-0.143	-0.165
Plasma_cells	-0.142	-0.055	-0.191	-0.236	-0.211	-0.226	-0.138	0.355	0.220	-0.079	0.457	-0.092
T_cells_CD8	0.412	0.451	0.460	0.331	0.355	0.438	0.391	0.419	0.697	0.370	0.105	0.431
T_cells_CD4_naive	NA	-0.265	-0.147	-0.225	-0.264	-0.031	-0.182	-0.304	-0.226	-0.409	-0.143	-0.376
T_cells_CD4_memory_resting	0.266	0.108	-0.006	0.045	0.059	-0.070	0.245	0.034	-0.085	0.231	0.253	0.118
T_cells_CD4_memory_activated	0.368	0.407	0.419	0.079	0.048	0.422	0.393	0.232	0.468	0.521	0.125	-0.056
T_cells_follicular_helper	0.114	0.149	0.120	0.193	0.179	0.118	0.068	0.313	0.212	0.065	0.186	0.134
T_cells_regulatory_(Tregs)	0.205	-0.034	0.074	0.081	0.102	0.071	0.332	0.346	0.379	0.081	0.375	0.189
T_cells_gamma_delta	0.006	0.086	0.051	-0.066	-0.083	0.037	-0.013	0.020	-0.044	-0.078	0.151	-0.150
NK_cells_resting	-0.060	-0.043	-0.113	-0.156	-0.116	-0.096	-0.053	-0.439	-0.427	-0.111	-0.463	0.009
NK_cells_activated	0.098	0.079	0.107	0.102	0.068	0.066	0.140	0.060	0.465	0.173	0.084	-0.030
Monocytes	-0.004	0.044	0.013	-0.017	-0.015	-0.013	0.064	-0.339	0.076	-0.125	-0.342	-0.011
Macrophages_M0	-0.074	-0.231	-0.295	-0.213	-0.201	-0.298	-0.146	-0.035	-0.344	-0.053	0.013	-0.150
Macrophages_M1	0.514	0.534	0.399	0.471	0.481	0.330	0.385	0.282	0.563	0.560	0.577	0.531
Macrophages_M2	-0.201	-0.164	-0.104	0.131	0.146	-0.089	-0.111	-0.200	-0.398	0.014	-0.364	0.206

Dendritic_cells_resting	0.125	0.147	0.182	0.083	0.100	0.207	0.001	0.191	0.227	0.044	0.369	0.141
Dendritic_cells_activated	-0.271	-0.273	-0.171	-0.099	-0.151	-0.035	-0.220	-0.119	-0.136	-0.285	-0.052	-0.304
Mast_cells_resting	-0.036	0.239	0.143	0.275	0.269	0.093	0.057	-0.496	-0.282	-0.057	-0.314	0.277
Mast_cells_activated	-0.149	-0.303	-0.301	-0.314	-0.270	-0.306	-0.215	0.123	-0.017	-0.072	-0.051	-0.182
Eosinophils	-0.106	-0.145	-0.093	-0.035	-0.020	-0.053	-0.128	-0.288	-0.238	-0.247	-0.325	0.014
Neutrophils	-0.194	-0.224	-0.209	0.125	0.127	-0.211	-0.127	-0.005	0.021	0.098	0.097	0.111

NA: Not available

Table 9 Correlation coefficient between TIGIT expression and immune related genes

Gene Type	Gene name	BLCA	READ	COAD	COADREAD	THCA	SKCM	LUSC	ESCA	STAD	STES	LUAD	LIHC
chemokine	CCL20	0.332	0.133	-0.073	-0.035	0.578	0.027	0.181	0.011	-0.023	-0.011	0.121	0.285
chemokine	CCL24	0.504	0.138	-0.086	-0.040	0.519	0.116	0.242	0.110	0.088	0.120	0.250	0.329
chemokine	CXCL2	0.481	0.245	0.114	0.140	0.459	0.177	0.194	0.167	0.053	0.117	0.148	0.119
chemokine	CXCL1	0.408	0.274	0.203	0.220	0.513	0.024	-0.001	-0.015	0.058	0.027	0.180	0.300
chemokine	CXCL3	0.456	0.170	0.098	0.113	0.441	0.072	0.161	0.049	0.069	0.107	0.136	0.351
chemokine	CXCL5	0.451	0.443	0.208	0.259	0.492	0.075	0.154	0.009	0.005	0.047	0.037	0.277
chemokine	CXCL6	0.318	0.359	0.275	0.287	0.395	-0.006	0.004	0.041	-0.047	-0.070	0.198	0.248
chemokine	CCL26	0.390	0.235	0.254	0.249	0.298	0.098	-0.059	0.071	0.110	0.101	0.258	0.266
chemokine	CXCL8	0.310	0.434	0.319	0.347	0.489	-0.002	-0.050	0.042	-0.017	-0.012	0.042	0.287
chemokine	CCL1	0.474	0.380	0.362	0.360	0.416	0.227	0.283	0.167	0.318	0.274	0.400	0.259
chemokine	CX3CL1	0.305	0.478	0.450	0.456	0.349	0.073	0.099	0.364	0.463	0.395	0.269	0.225
chemokine	CCL7	0.549	0.557	0.431	0.461	0.548	0.219	0.317	0.297	0.150	0.202	0.214	0.301
chemokine	CCL11	0.445	0.496	0.488	0.483	0.505	0.127	0.337	0.279	0.389	0.385	0.406	0.329

chemokine	CCL27	-0.099	-0.086	-0.022	-0.036	-0.067	0.236	-0.040	0.034	-0.082	-0.073	-0.090	0.054
chemokine	CXCL17	-0.026	0.002	0.083	0.069	0.513	0.001	0.075	0.081	0.037	0.043	-0.007	0.178
chemokine	CCL25	0.385	0.161	0.300	0.270	0.151	0.424	0.367	0.153	0.231	0.242	0.443	0.070
chemokine	CCL14	0.260	0.249	0.406	0.371	-0.151	0.203	0.382	0.278	0.195	0.235	0.246	-0.121
chemokine	CCL16	0.052	0.174	0.137	0.144	-0.118	0.125	0.267	0.251	0.058	0.143	0.198	-0.166
chemokine	CCL15	-0.179	-0.132	-0.290	-0.259	0.141	0.181	0.259	-0.005	0.025	0.101	0.143	-0.140
chemokine	CCL28	0.234	0.227	0.108	0.138	-0.093	0.008	0.063	0.090	-0.108	-0.030	0.192	0.136
chemokine	CXCL14	0.003	0.070	-0.058	-0.041	0.423	0.122	-0.034	0.057	0.043	0.002	0.229	0.377
chemokine	CCL5	0.684	0.781	0.836	0.824	0.820	0.879	0.755	0.749	0.834	0.810	0.803	0.847
chemokine	CXCL9	0.805	0.800	0.820	0.816	0.847	0.897	0.714	0.809	0.758	0.781	0.785	0.763
chemokine	CXCL13	0.782	0.777	0.796	0.792	0.830	0.889	0.617	0.769	0.780	0.783	0.714	0.584
chemokine	CCL4	0.788	0.784	0.746	0.754	0.755	0.874	0.720	0.657	0.667	0.676	0.738	0.765
chemokine	XCL2	0.556	0.448	0.653	0.607	0.665	0.792	0.577	0.609	0.633	0.639	0.570	0.570
chemokine	CXCL10	0.749	0.743	0.750	0.749	0.832	0.807	0.622	0.683	0.659	0.666	0.679	0.574
chemokine	CXCL11	0.694	0.677	0.572	0.595	0.805	0.772	0.579	0.675	0.586	0.619	0.644	0.579
chemokine	CCL18	0.682	0.652	0.589	0.601	0.564	0.081	0.495	0.362	0.425	0.415	0.455	0.522
chemokine	CCL21	0.497	0.482	0.511	0.501	0.469	0.247	0.508	0.383	0.418	0.423	0.443	0.371
chemokine	CCL17	0.284	0.389	0.545	0.506	0.570	0.188	0.335	0.336	0.445	0.403	0.313	0.370
chemokine	CCL23	0.582	0.546	0.513	0.521	0.617	0.286	0.442	0.295	0.390	0.383	0.292	0.317
chemokine	CXCL16	0.529	0.541	0.379	0.414	0.608	0.510	0.409	0.201	0.255	0.273	0.120	0.237
chemokine	CCL2	0.575	0.678	0.571	0.589	0.603	0.368	0.454	0.440	0.389	0.429	0.375	0.444
chemokine	CXCL12	0.453	0.674	0.481	0.518	0.514	0.460	0.475	0.294	0.346	0.359	0.438	0.333
chemokine	XCL1	0.299	0.310	0.500	0.447	0.674	0.798	0.059	0.181	0.405	0.292	0.392	0.519
chemokine	CCL22	0.506	0.674	0.717	0.703	0.748	0.388	0.463	0.497	0.640	0.587	0.475	0.554
chemokine	CCL19	0.625	0.573	0.691	0.663	0.781	0.492	0.644	0.561	0.629	0.622	0.693	0.531
chemokine	CCL3	0.711	0.634	0.548	0.568	0.534	0.531	0.531	0.403	0.392	0.416	0.485	0.597

chemokine	CCL8	0.640	0.682	0.603	0.619	0.505	0.663	0.449	0.491	0.418	0.461	0.448	0.525
chemokine	CCL13	0.601	0.683	0.598	0.615	0.618	0.408	0.385	0.383	0.459	0.436	0.352	0.515
receptor	CCR4	0.721	0.839	0.773	0.786	0.809	0.684	0.754	0.691	0.773	0.755	0.710	0.606
receptor	CCR7	0.150	0.648	0.786	0.755	0.841	0.705	0.754	0.649	0.704	0.697	0.770	0.670
receptor	CCR1	0.593	0.750	0.686	0.698	0.705	0.630	0.636	0.679	0.638	0.664	0.517	0.655
receptor	CCR2	0.667	0.818	0.719	0.736	0.796	0.764	0.716	0.654	0.720	0.708	0.677	0.656
receptor	CCR8	0.715	0.720	0.733	0.725	0.647	0.575	0.707	0.718	0.721	0.720	0.727	0.601
receptor	XCR1	0.230	0.522	0.637	0.610	0.785	0.703	0.547	0.457	0.646	0.591	0.513	0.663
receptor	CXCR3	0.808	0.197	0.360	0.324	0.696	0.910	0.846	0.662	0.771	0.730	0.749	0.807
receptor	CCR5	0.810	0.895	0.901	0.899	0.861	0.882	0.843	0.832	0.870	0.857	0.836	0.834
receptor	CXCR6	0.793	0.855	0.870	0.867	0.849	0.890	0.829	0.773	0.848	0.830	0.841	0.822
receptor	CXCR1	0.310	0.439	0.416	0.419	0.129	0.045	0.082	0.121	0.146	0.149	0.097	0.231
receptor	CXCR2	0.001	0.521	0.393	0.416	0.470	0.130	0.117	0.054	0.111	0.074	0.208	0.282
receptor	CCR10	0.046	0.306	0.439	0.403	0.047	0.101	0.212	0.230	0.323	0.317	0.083	0.303
receptor	CXCR5	0.208	0.105	0.228	0.204	0.283	0.356	0.276	0.169	0.298	0.234	0.299	0.211
receptor	CCR9	0.136	0.372	0.333	0.340	0.438	0.209	0.233	0.272	0.318	0.330	0.286	0.242
receptor	CCR3	0.093	0.526	0.443	0.459	0.350	0.517	0.261	0.268	0.336	0.331	0.221	0.368
receptor	CX3CR1	0.339	0.531	0.435	0.449	0.274	0.462	0.396	0.436	0.366	0.410	0.209	0.319
receptor	CCR6	0.614	0.142	0.309	0.271	0.637	0.502	0.526	0.292	0.366	0.367	0.533	0.383
receptor	CXCR4	0.618	0.644	0.662	0.658	0.623	0.583	0.608	0.514	0.587	0.580	0.656	0.661
MHC	HLA-G	0.269	0.209	0.143	0.158	0.538	0.278	0.290	0.270	0.131	0.183	0.314	0.301
MHC	HLA-DMB	0.759	0.730	0.794	0.780	0.384	0.772	0.679	0.637	0.684	0.680	0.518	0.741
MHC	HLA-DOB	0.681	0.617	0.682	0.668	0.818	0.715	0.580	0.569	0.649	0.612	0.575	0.630
MHC	HLA-DOA	0.658	0.824	0.814	0.815	0.842	0.679	0.675	0.706	0.767	0.756	0.581	0.742
MHC	HLA-DPA1	0.743	0.829	0.812	0.815	0.845	0.760	0.694	0.682	0.697	0.702	0.536	0.719
MHC	HLA-DPB1	0.758	0.815	0.817	0.816	0.860	0.791	0.690	0.718	0.745	0.744	0.544	0.731

MHC	HLA-DRA	0.765	0.826	0.826	0.825	0.849	0.777	0.683	0.668	0.700	0.701	0.523	0.729
MHC	HLA-DMA	0.618	0.596	0.721	0.694	0.801	0.728	0.677	0.533	0.572	0.577	0.408	0.592
MHC	HLA-DQA1	0.755	0.748	0.752	0.752	0.774	0.671	0.602	0.646	0.713	0.704	0.555	0.688
MHC	HLA-DRB1	0.684	0.720	0.720	0.719	0.754	0.740	0.601	0.598	0.645	0.643	0.466	0.666
MHC	HLA-E	0.633	0.648	0.571	0.586	0.674	0.656	0.613	0.583	0.614	0.594	0.559	0.483
MHC	TAP1	0.691	0.599	0.572	0.577	0.814	0.713	0.564	0.521	0.500	0.480	0.630	0.600
MHC	HLA-F	0.603	0.382	0.458	0.443	0.762	0.716	0.620	0.513	0.419	0.460	0.598	0.431
MHC	B2M	0.657	0.597	0.546	0.558	0.716	0.668	0.616	0.535	0.461	0.492	0.561	0.463
MHC	HLA-B	0.629	0.444	0.439	0.438	0.717	0.752	0.638	0.515	0.358	0.409	0.608	0.454
MHC	HLA-DQA2	0.541	0.594	0.470	0.489	0.729	0.570	0.469	0.443	0.442	0.445	0.371	0.467
MHC	HLA-DQB1	0.575	0.283	0.484	0.445	0.489	0.584	0.432	0.470	0.523	0.520	0.354	0.556
MHC	TAP2	0.579	0.472	0.446	0.450	0.610	0.481	0.324	0.379	0.389	0.336	0.552	0.386
MHC	TAPBP	0.519	0.330	0.395	0.383	0.459	0.529	0.385	0.372	0.486	0.426	0.430	0.343
MHC	HLA-A	0.576	0.394	0.300	0.311	0.676	0.507	0.499	0.439	0.294	0.341	0.457	0.477
MHC	HLA-C	0.557	0.453	0.485	0.477	0.648	0.613	0.501	0.450	0.386	0.416	0.474	0.315
Immunoinhibitor	IL10RB	0.149	0.269	0.044	0.099	0.272	0.247	0.086	0.015	0.051	0.058	0.155	0.183
Immunoinhibitor	TGFB1	0.195	0.552	0.597	0.588	0.261	0.132	0.039	0.227	0.462	0.331	0.411	0.446
Immunoinhibitor	KDR	0.187	0.656	0.494	0.523	-0.242	-0.089	0.366	0.285	0.257	0.282	0.108	0.104
Immunoinhibitor	TGFBR1	0.391	0.448	0.355	0.367	0.320	0.046	0.111	0.218	0.215	0.177	0.161	0.270
Immunoinhibitor	PVRL2	0.171	-0.030	0.182	0.136	-0.193	-0.043	0.122	-0.005	0.016	0.028	0.046	0.082
Immunoinhibitor	VTCN1	-0.071	-0.008	0.081	0.064	0.450	0.079	-0.160	-0.019	-0.152	-0.149	0.031	0.196
Immunoinhibitor	TIGIT	1.000	1.000	1.000	1.000	1.000	1.000	1.000	1.000	1.000	1.000	1.000	1.000
Immunoinhibitor	CTLA4	0.842	0.733	0.842	0.816	0.829	0.578	0.822	0.835	0.684	0.724	0.855	0.814
Immunoinhibitor	LAG3	0.757	0.716	0.780	0.766	0.772	0.881	0.762	0.780	0.766	0.773	0.700	0.682
Immunoinhibitor	CD96	0.340	0.866	0.886	0.881	0.698	0.870	0.736	0.844	0.859	0.854	0.749	0.800
Immunoinhibitor	PDCD1	0.825	0.795	0.815	0.809	0.650	0.896	0.818	0.809	0.797	0.807	0.817	0.745

Immunoinhibitor	CD244	0.476	0.665	0.733	0.718	0.736	0.645	0.669	0.661	0.728	0.716	0.674	0.676
Immunoinhibitor	PDCD1LG2	0.736	0.799	0.770	0.775	0.767	0.678	0.556	0.618	0.751	0.706	0.702	0.702
Immunoinhibitor	CSF1R	0.680	0.762	0.729	0.736	0.729	0.652	0.623	0.619	0.612	0.628	0.580	0.653
Immunoinhibitor	HAVCR2	0.794	0.801	0.789	0.792	0.818	0.814	0.699	0.754	0.717	0.739	0.639	0.716
Immunoinhibitor	LGALS9	0.402	0.380	0.306	0.326	0.719	0.560	0.578	0.255	0.391	0.375	0.421	0.568
Immunoinhibitor	CD160	0.410	0.529	0.444	0.466	0.387	0.382	0.495	0.356	0.380	0.382	0.463	0.479
Immunoinhibitor	KIR2DL1	0.455	0.389	0.482	0.463	0.191	0.590	0.374	0.427	0.442	0.457	0.271	0.213
Immunoinhibitor	KIR2DL3	0.553	0.421	0.503	0.486	0.389	0.631	0.470	0.570	0.542	0.568	0.347	0.366
Immunoinhibitor	ADORA2A	0.586	0.679	0.768	0.744	0.298	0.722	0.575	0.553	0.585	0.591	0.510	0.354
Immunoinhibitor	BTLA	0.729	0.569	0.623	0.612	0.821	0.774	0.738	0.704	0.734	0.732	0.796	0.707
Immunoinhibitor	IDO1	0.704	0.758	0.768	0.763	0.650	0.803	0.543	0.748	0.667	0.689	0.542	0.587
Immunoinhibitor	CD274	0.588	0.653	0.726	0.709	0.592	0.631	0.345	0.436	0.564	0.502	0.573	0.539
Immunoinhibitor	IL10	0.624	0.567	0.617	0.602	0.585	0.511	0.479	0.502	0.482	0.495	0.481	0.537
Immunostimulator	CD276	0.251	0.394	0.326	0.342	0.330	-0.097	-0.058	0.068	0.113	0.061	0.032	0.261
Immunostimulator	PVR	0.241	0.218	-0.038	0.019	-0.116	-0.053	-0.079	-0.025	-0.031	-0.044	0.090	-0.027
Immunostimulator	ULBP1	0.143	0.296	-0.151	-0.031	-0.028	-0.013	-0.075	-0.064	-0.099	-0.150	0.137	0.197
Immunostimulator	IL6R	0.339	0.632	0.563	0.579	0.591	0.042	0.231	0.214	0.376	0.295	0.092	-0.115
Immunostimulator	TNFRSF14	0.147	0.299	0.295	0.297	0.151	0.128	0.519	0.377	0.452	0.445	0.376	0.342
Immunostimulator	TNFSF13	0.312	0.311	0.356	0.348	0.116	0.326	0.441	0.139	0.228	0.243	0.164	0.407
Immunostimulator	ICOSLG	0.253	0.265	0.202	0.216	0.214	0.194	0.279	0.104	0.221	0.200	0.292	0.118
Immunostimulator	TNFSF15	0.038	0.377	0.043	0.122	0.207	0.334	0.387	0.128	0.151	0.174	0.058	0.344
Immunostimulator	IL6	0.546	0.486	0.408	0.425	0.438	0.069	0.163	0.214	0.166	0.181	0.279	0.321
Immunostimulator	TNFSF18	0.406	0.593	0.391	0.430	0.492	0.315	0.079	0.183	0.357	0.273	0.283	0.383
Immunostimulator	NT5E	0.308	0.285	0.180	0.203	0.360	0.149	0.116	0.022	0.094	0.068	0.154	0.130
Immunostimulator	TNFSF9	0.336	-0.052	0.216	0.166	0.533	0.133	0.003	0.011	0.194	0.123	0.089	0.438
Immunostimulator	RAET1E	-0.040	0.475	0.233	0.294	0.307	-0.022	0.010	-0.088	0.029	-0.087	0.185	0.160

Immunostimulator	TNFRSF25	0.007	0.123	0.080	0.087	0.362	0.313	0.094	0.199	0.082	0.035	0.283	0.279
Immunostimulator	BTNL2	-0.009	0.363	0.132	0.189	0.055	0.216	0.118	0.294	0.246	0.250	0.253	0.103
Immunostimulator	HHLA2	0.265	0.258	0.014	0.054	0.246	0.338	0.186	0.022	-0.012	0.057	0.144	0.277
Immunostimulator	TNFRSF13C	0.346	0.436	0.528	0.509	0.597	0.125	0.299	0.457	0.504	0.484	0.599	0.590
Immunostimulator	CXCR4	0.618	0.644	0.662	0.658	0.623	0.583	0.608	0.514	0.587	0.580	0.656	0.661
Immunostimulator	TMIGD2	0.262	0.424	0.349	0.368	0.582	0.616	0.574	0.379	0.502	0.473	0.563	0.395
Immunostimulator	CD70	0.650	0.313	0.479	0.445	0.629	0.428	0.407	0.356	0.419	0.393	0.563	0.707
Immunostimulator	C10orf54	0.398	0.685	0.584	0.606	0.217	0.426	0.544	0.284	0.414	0.384	0.544	0.461
Immunostimulator	TNFRSF8	0.632	0.740	0.786	0.773	0.313	0.643	0.602	0.582	0.583	0.588	0.651	0.755
Immunostimulator	MICB	0.474	0.424	0.507	0.489	0.652	0.225	0.419	0.411	0.433	0.407	0.484	0.370
Immunostimulator	TNFSF4	0.568	0.639	0.617	0.621	0.535	0.340	0.543	0.565	0.526	0.551	0.515	0.217
Immunostimulator	ENTPD1	0.572	0.693	0.659	0.660	0.028	0.414	0.597	0.576	0.498	0.532	0.549	0.526
Immunostimulator	CD40	0.361	0.596	0.590	0.589	0.487	0.427	0.385	0.506	0.537	0.537	0.563	0.310
Immunostimulator	TNFRSF4	0.626	0.556	0.640	0.620	0.233	0.455	0.516	0.658	0.608	0.638	0.460	0.485
Immunostimulator	TNFRSF18	0.610	0.601	0.766	0.731	0.713	0.644	-0.007	0.240	0.501	0.310	0.346	0.678
Immunostimulator	CXCL12	0.453	0.674	0.481	0.518	0.514	0.460	0.475	0.294	0.346	0.359	0.438	0.333
Immunostimulator	TMEM173	0.486	0.338	0.306	0.314	0.439	-0.019	0.317	0.294	0.426	0.368	0.171	0.507
Immunostimulator	TNFSF14	0.649	0.802	0.783	0.787	0.748	0.770	0.627	0.534	0.640	0.615	0.457	0.104
Immunostimulator	CD27	0.835	0.818	0.848	0.841	0.298	0.931	0.781	0.779	0.808	0.805	0.772	0.904
Immunostimulator	CD48	0.803	0.877	0.839	0.846	0.887	0.855	0.727	0.813	0.806	0.807	0.697	0.842
Immunostimulator	ICOS	0.863	0.887	0.882	0.882	0.893	0.813	0.814	0.839	0.810	0.807	0.874	0.850
Immunostimulator	TNFSF13B	0.752	0.677	0.753	0.735	0.824	0.624	0.703	0.738	0.746	0.753	0.679	0.672
Immunostimulator	KLRK1	0.718	0.565	0.806	0.753	0.776	0.880	0.799	0.816	0.750	0.776	0.804	0.785
Immunostimulator	LTA	0.762	0.861	0.768	0.785	0.819	0.849	0.808	0.687	0.830	0.790	0.809	0.787
Immunostimulator	TNFRSF9	0.777	0.866	0.830	0.838	0.845	0.929	0.718	0.685	0.792	0.762	0.689	0.669
Immunostimulator	CD80	0.736	0.737	0.713	0.715	0.677	0.639	0.681	0.692	0.660	0.669	0.636	0.673

Immunostimulator	CD86	0.786	0.808	0.785	0.789	0.839	0.758	0.656	0.670	0.693	0.694	0.645	0.765
Immunostimulator	IL2RA	0.806	0.816	0.833	0.827	0.739	0.606	0.677	0.707	0.743	0.736	0.700	0.736
Immunostimulator	CD28	0.768	0.783	0.764	0.766	0.838	0.529	0.723	0.765	0.727	0.746	0.723	0.641
Immunostimulator	KLRC1	0.509	0.468	0.543	0.528	0.612	0.750	0.612	0.675	0.584	0.608	0.478	0.547
Immunostimulator	CD40LG	0.610	0.614	0.607	0.604	0.776	0.672	0.746	0.551	0.617	0.606	0.667	0.608
Immunostimulator	TNFRSF13B	0.595	0.524	0.643	0.616	0.781	0.566	0.636	0.557	0.633	0.623	0.622	0.654
Immunostimulator	TNFRSF17	0.582	0.584	0.654	0.637	0.653	0.632	0.592	0.523	0.587	0.581	0.524	0.659

Table 10 Correlation between TIGIT expression and TMB

Cancer Type	Correlation coefficient	P value
COAD	R=0.26	P=0.00000704129303332121
COADREAD	R=0.25	P=6.40696539541314e-7
STES	R=0.09	P=0.0243863280748041
READ	R=0.22	P=0.0380099918857127
BLCA	R=0.11	P=0.0247453791498405
STAD	R=0.08	P=0.119
ESCA	R=0.15	P<0.001
LUAD	R=0.029	P=0.512
LUSC	R=-0.001	P=0.986
LIHC	R=-0.060	P=0.256
THCA	R=0.014	P=0.755
SKCM	R=0.100	P=0.315

Table 11 Correlation between TIGIT expression and MSI

Cancer Type	Correlation coefficient	P value
COAD	R=0.26	P=0.0000058
COADREAD	R=0.24	P=0.0000020
STES	R=0.09	P=0.024
THCA	R=0.041	P=0.367
BLCA	R=-0.048	P=0.335
SKCM	R=-0.044	P=0.662
STAD	R=0.089	P=0.072
READ	R=0.108	P=0.315
ESCA	R=-0.059	P=0.431
LIHC	R=-0.050	P=0.344
LUSC	R=-0.051	P=0.262
LUAD	R=0.056	P=0.207

A

B

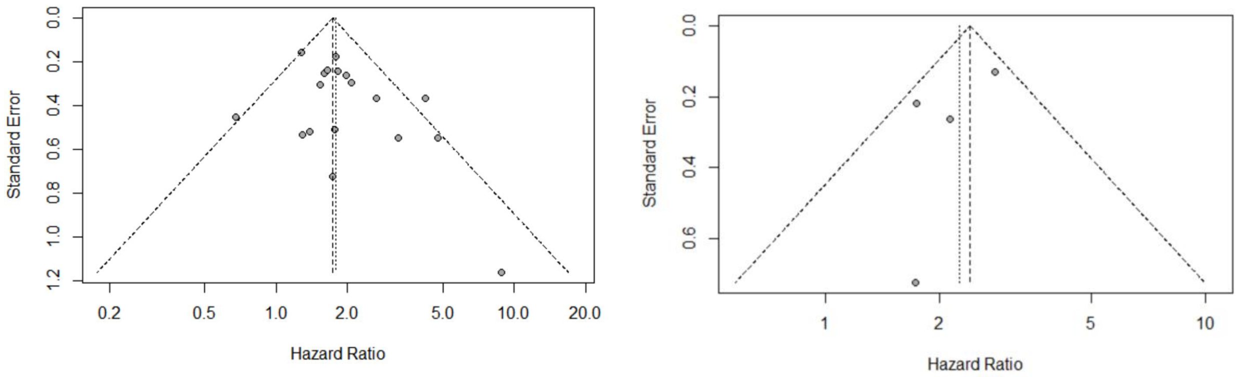

Figure 1 Begg's forest plots (A.OS B. RFS)

A B

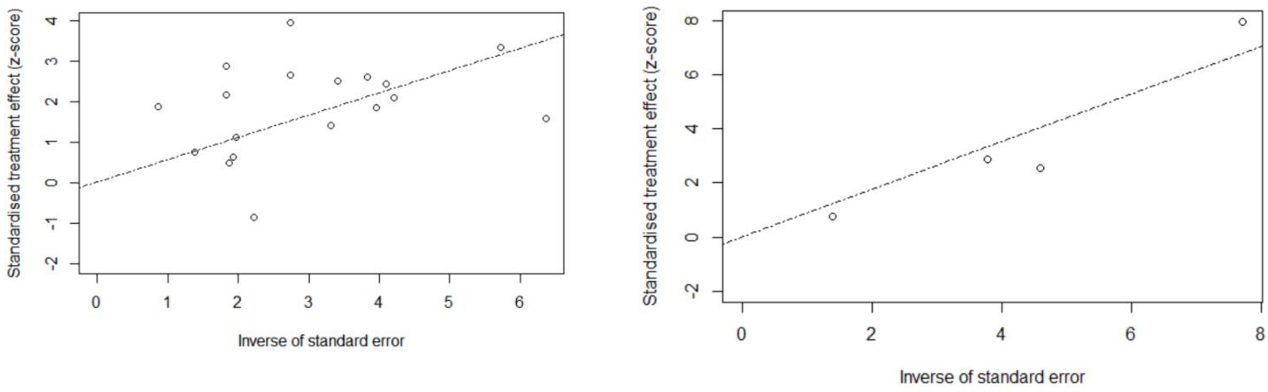

Figure 2 Egger's regression asymmetry plot (A.OS B. RFS)

A

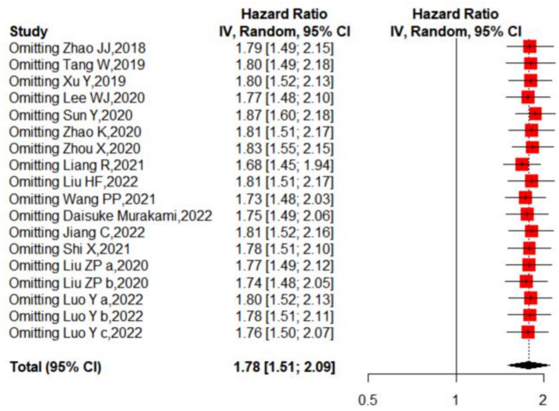

B

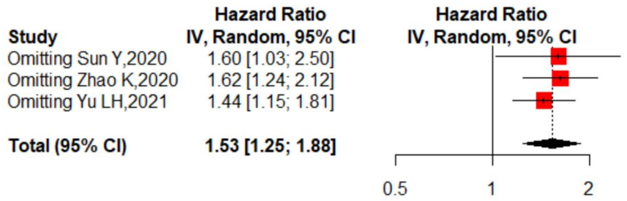

C

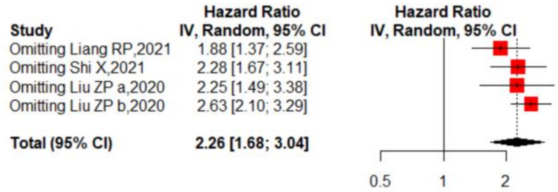

D

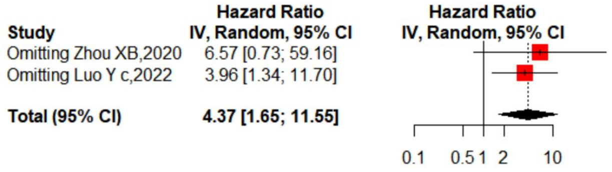

Figure 3 Sensitivity analysis plot(A.OS B.PFS C.RFS D.DFS)

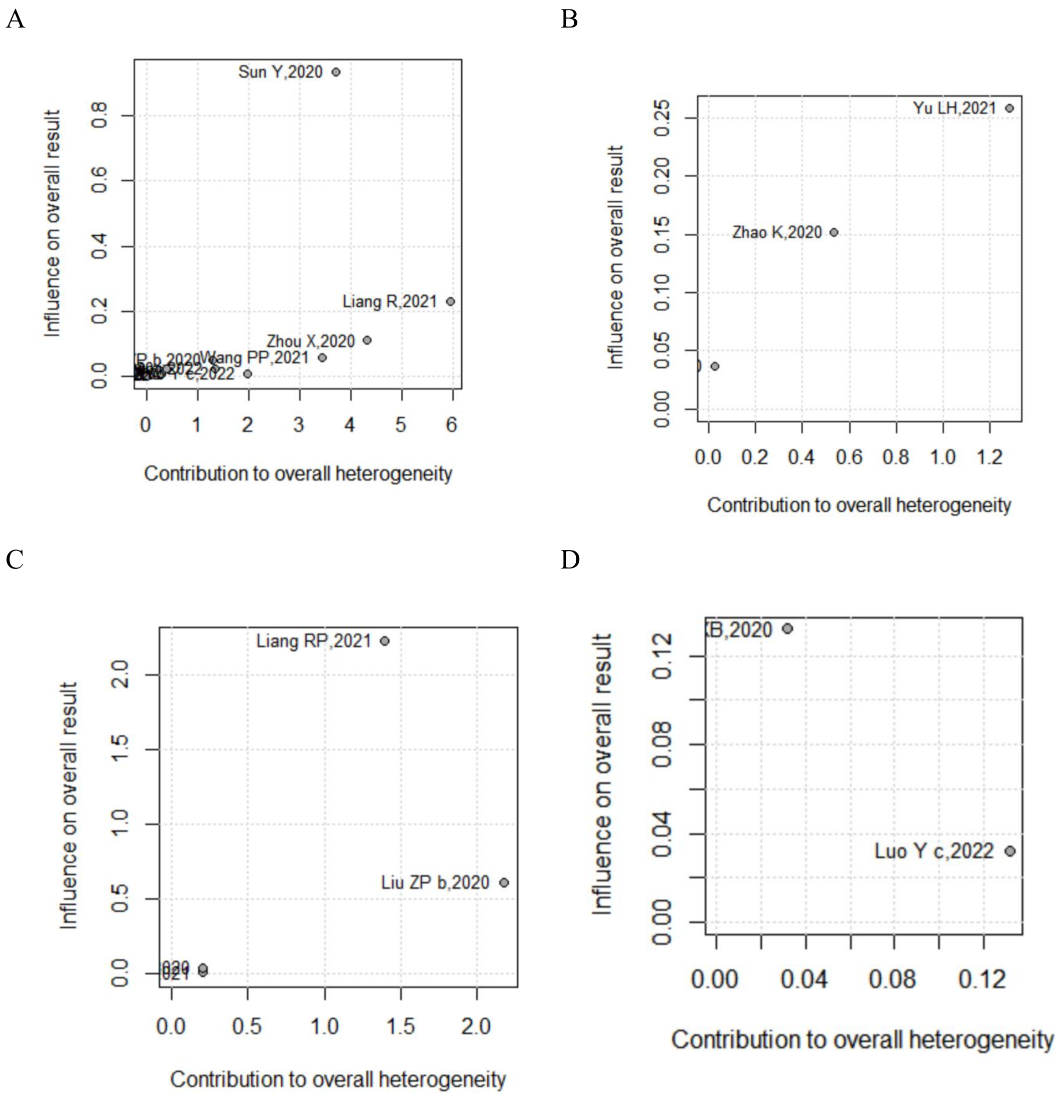

Figure 4 Baujat plot for heterogeneity analysis(A.OS B.PFS C.RFS D.DFS)

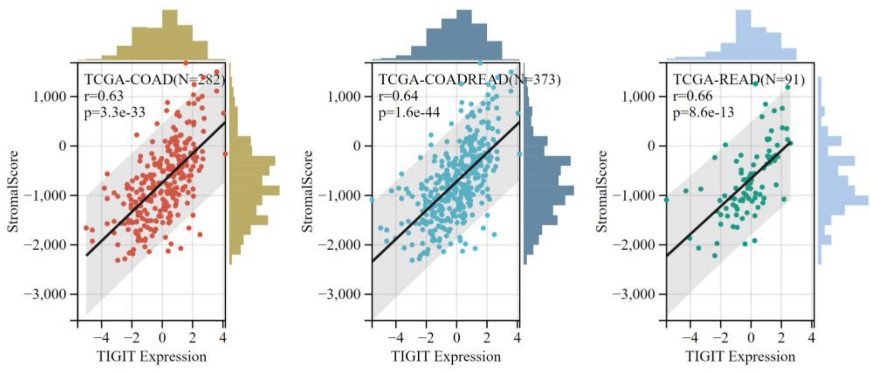

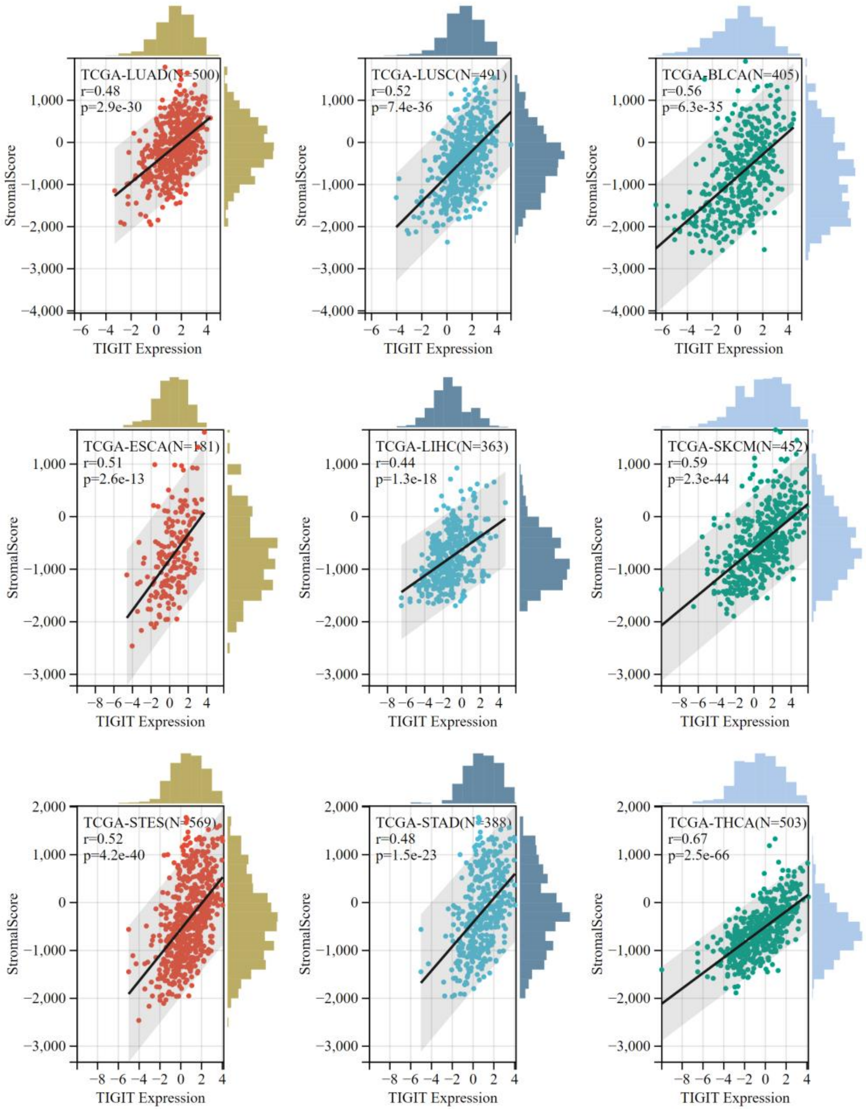

Figure 5 Correlation between TIGIT expression and StromalScore

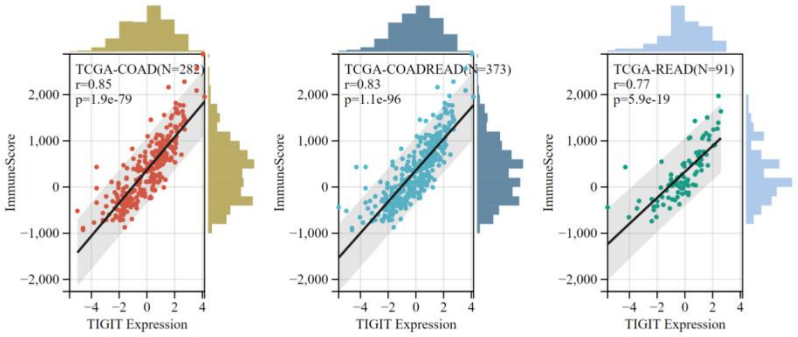

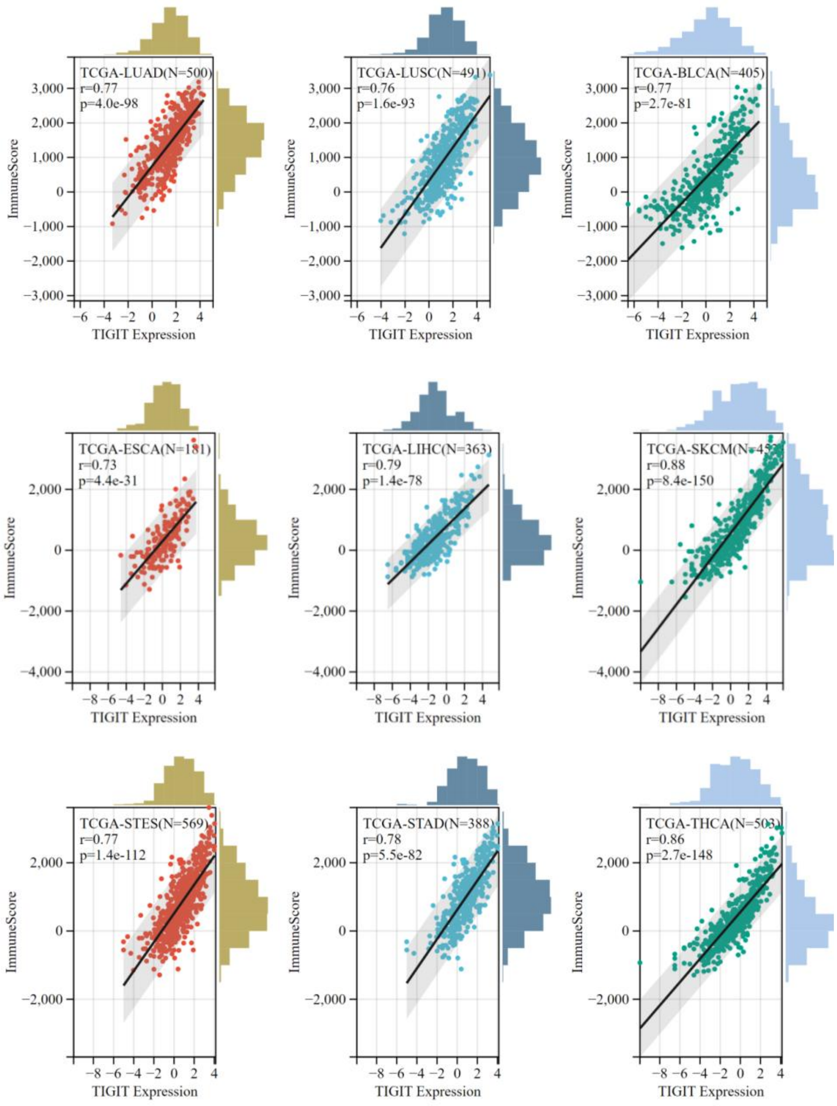

Figure 6 Correlation between TIGIT expression and ImmuneScore

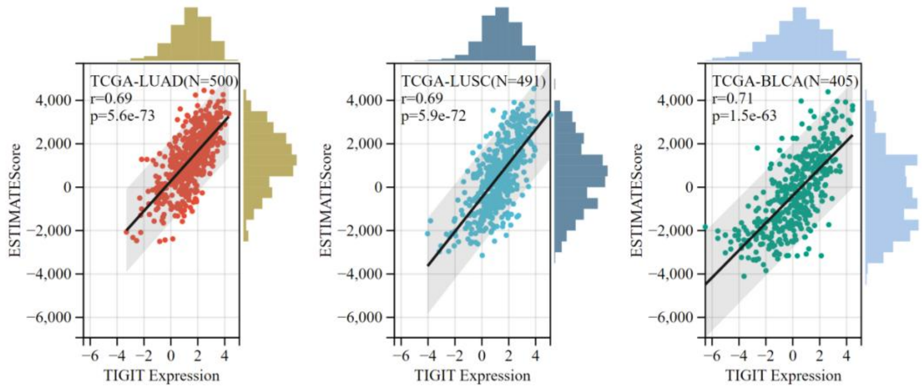

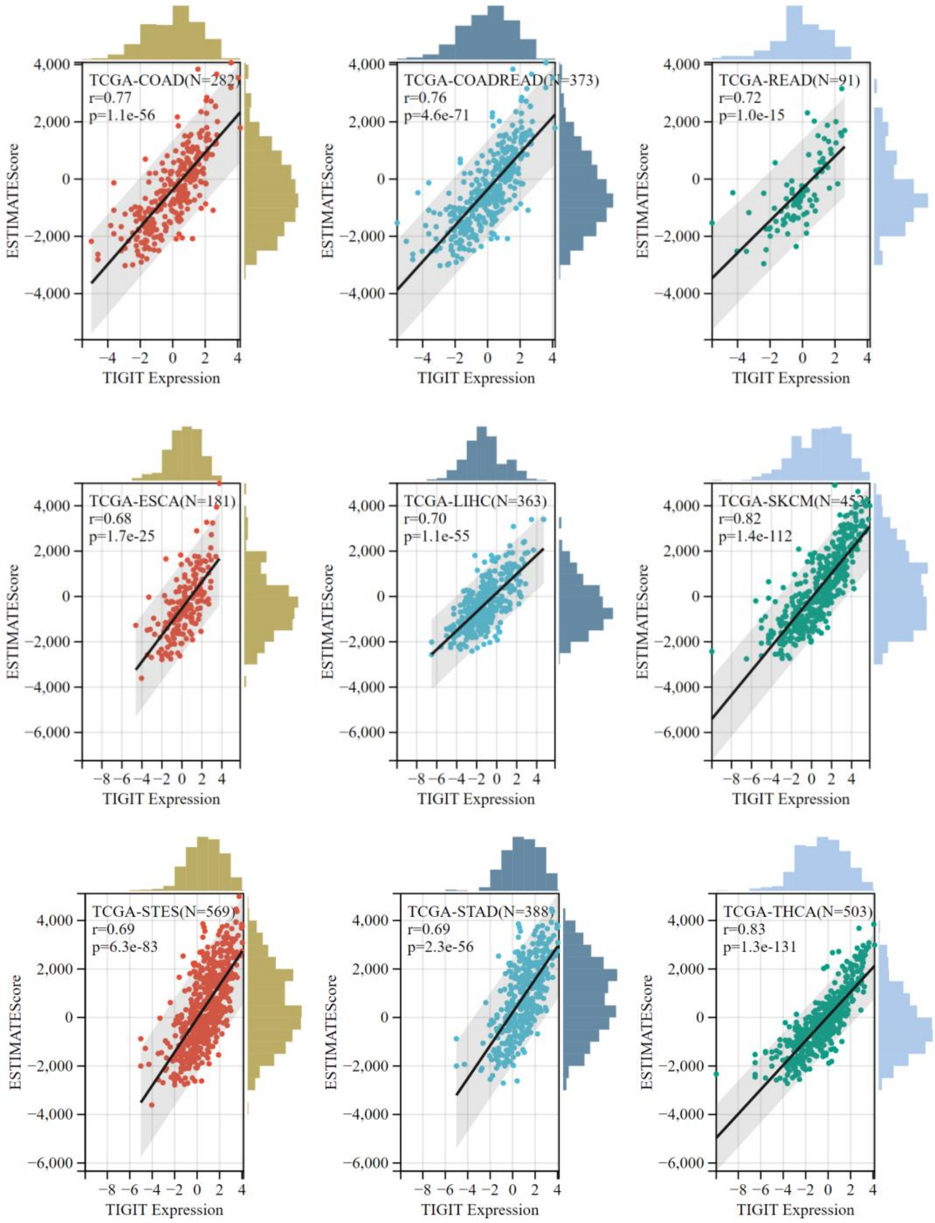

Figure 7 Correlation between TIGIT expression and ESTIMATE score
